# The allosteric IDH1 inhibitor ivosidenib overcomes chemoresistance in intrahepatic cholangiocarcinoma models expressing wild-type IDH1

**DOI:** 10.1172/JCI199730

**Published:** 2026-03-17

**Authors:** Xiuxian Li, Zhixiao Song, Shusheng Lin, Man Luo, Shaoru Liu, Yang Liu, Fapeng Zhang, Leibo Xu, Chao Liu, Honghua Zhang

**Affiliations:** 1 Department of Biliary-Pancreatic Surgery,; 2 Guangzhou Key Laboratory of Precise Diagnosis and Treatment of Biliary Tract Cancer,; 3 Guangdong Provincial Key Laboratory of Malignant Tumor Epigenetics and Gene Regulation, Guangdong-Hong Kong Joint Laboratory for RNA medicine, and; 4 Department of Ultrasound, Sun Yat-sen Memorial Hospital, Sun Yat-sen University, Guangzhou, Guangdong Province, China.; 5 Department of Liver Surgery, Sun Yat-sen University Cancer Center, Sun Yat-sen University, Guangzhou, Guangdong Province, China.

**Keywords:** Hepatology, Metabolism, Oncology, Cancer gene therapy, Liver cancer

## Abstract

Gemcitabine-based chemotherapy is the standard treatment regimen for advanced intrahepatic cholangiocarcinoma (iCCA), but the frequent presence of chemoresistance limits its efficacy. Here, we identified isocitrate dehydrogenase 1 (IDH1) as the crucial target that confers chemoresistance of iCCA to gemcitabine using a druggable CRISPR/Cas9 library. The positive association between IDH1 expression and chemoresistance was revealed in a gemcitabine-treated iCCA cohort and with cell-based drug sensitivity assays. Utilizing patient-derived organoids, cell line–derived xenografts, and patient-derived xenografts, we demonstrated that IDH1 knockdown or IDH1 pharmacological inhibition facilitated gemcitabine efficacy in these preclinical iCCA models carrying wild-type IDH1 (wtIDH1). Mechanistically, wtIDH1 oxidizes isocitrate to generate α-ketoglutarate and NNADPH, thereby creating a mechanism to manage the oxidative stress induced by gemcitabine, maintaining cellular redox homeostasis, and, ultimately, leading to chemoresistance to gemcitabine. Significantly, ivosidenib, the FDA-approved allosteric IDH1 inhibitor, demonstrated synergistic antitumor efficacy with gemcitabine in wtIDH1 preclinical iCCA models through boosting intracellular oxidative stress under physiological conditions. The low level of Mg^2+^, an ion that competitively hinders binding of ivosidenib on wtIDH1, in the iCCA tumor microenvironment contributed to the expanded therapeutic window for use of ivosidenib in patients with iCCA. Our work revealed the potency of combining targeting IDH1 and chemotherapy against wtIDH1 iCCA and other tumors.

## Abstract 2

The FDA-approved drug ivosidenib originally designed for mutant IDH1 is also effective in intrahepatic cholangiocarcinoma expressing wild-type IDH1

## Graphical abstract

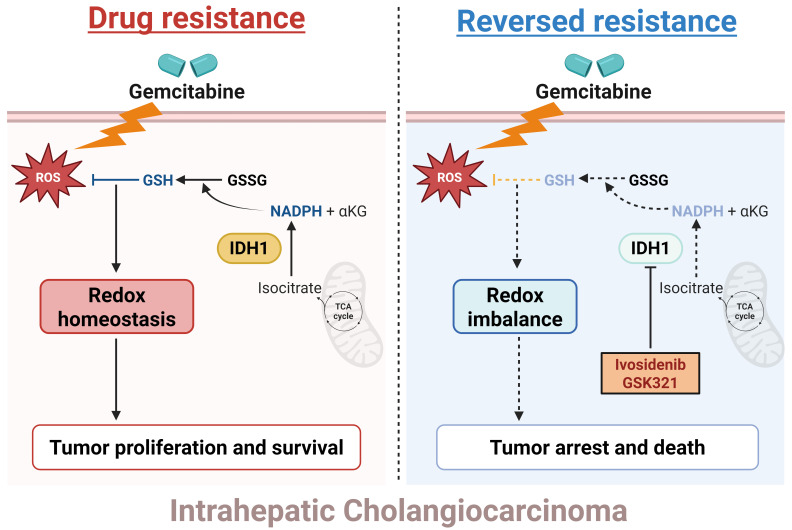

## Introduction

Intrahepatic cholangiocarcinoma (iCCA) is the second most common primary liver cancer, characterized by low eligibility for radical resection and a poor prognosis, with a median overall survival (OS) of approximately 10 months (1–3). In recent years, several molecular therapies have emerged as promising treatment options for advanced iCCA, including drugs targeting FGFR2 fusions and isocitrate dehydrogenase 1 (IDH1) mutations (4–6). The FDA-approved FGFR2 inhibitor pemigatinib and the allosteric IDH1 inhibitor ivosidenib have demonstrated efficacy in patients with advanced iCCA harboring these specific genetic alterations. However, the low prevalence of these genetic alterations limits the broader clinical application of these targeted therapies. Compelling results from the TOPAZ-1 and KEYNOTE-966 global phase III trials have demonstrated that combining anti–PD-L1/PD-1 immunotherapy with standard chemotherapy improves outcomes in advanced iCCA (7–9). Nevertheless, the improvement in median OS remains modest, with an increase of no more than 2 months. Despite these advancements, systemic chemotherapy remains the only first-line regimen currently recommended for patients with advanced iCCA (10–12), but the efficacy during the initial treatment phase is inefficient in more than 60% of patients with iCCA (13). Thus, exploring the molecular mechanisms of chemoresistance and identifying potential approaches to reverse chemoresistance are crucial for improving long-term prognosis in iCCA.

The molecular basis of chemoresistance in iCCA is multifaceted, involving aberrant signaling pathways, such as NF-κB and TGF-β signaling (14, 15); gene alterations, including PTEN deficiency (16, 17); and cellular self-protective responses to defend against apoptosis, ferroptosis, and pyroptosis (18–20). In addition to these intrinsic molecular abnormalities within cancer cells, the hypovascular nature of solid tumors also contributes to chemoresistance by impeding drug delivery due to poor blood supply, which also creates a nutrient-poor tumor microenvironment (TME) and raises survival stress and the dependence of tumor cells on antioxidant defense (21, 22). Therefore, targeting crucial pathways involved in intracellular antioxidative stress represents an attractive therapeutic alternative, addressing a biological vulnerability of iCCA in the context of nutrient-poor TME. Gemcitabine (GEM) is the most commonly used chemotherapy drug for iCCA; it inhibits tumor proliferation by blocking DNA synthesis and inducing excessive intracellular oxidative stress (23). Drawing on the “two-hit hypothesis,” which posits that tumorigenesis occurs owing to sequential gene alterations (24), we speculate that multimodal approaches are necessary to surpass the cellular antioxidative self-defense threshold, thereby ultimately inducing cell death.

The genetic landscape of iCCA has been extensively explored in comprehensive molecular profiling studies (25–29) that unveil a wide range of actionable genetic vulnerabilities. Among the heterogeneous genetic alterations observed in iCCA, IDH1 mutation is a prominent hotspot, and its FDA-approved allosteric inhibitor ivosidenib has shown encouraging therapeutic efficacy in advanced iCCA (5, 6). However, the clinical application of ivosidenib is limited by the low prevalence of IDH1 mutations in iCCA. On the other hand, wild-type IDH1 (wtIDH1) plays a crucial role in cellular metabolism. As an important enzyme in the TCA cycle, wtIDH1 catalyzes the oxidative decarboxylation of isocitrate to produce CO_2_ and α-ketoglutarate (αKG), which is crucial for maintaining cellular redox homeostasis in a NADP^+^-dependent way (30). Given that most chemotherapy drugs are strong prooxidants, targeting wtIDH1 may be a potential approach to exacerbate redox imbalance and induce cytotoxic tumor cell death when combined with chemotherapy. Consistent with this speculation, preclinical studies have shown that combination of ivosidenib and chemotherapy exhibits profound efficacy in IDH1-mutated acute myeloid leukemia and wtIDH1 pancreatic ductal adenocarcinoma models (31, 32). Therefore, to expand the population of patients with iCCA who could benefit from IDH1-targeted therapies, regardless of their IDH1 mutation status, further mechanistic and translational research is warranted for improving clinical outcomes in iCCA.

In this study, we investigated the role of wtIDH1 in chemoresistance and its therapeutic vulnerability in iCCA. Using a customized druggable CRISPR/Cas9 library and analysis of a clinicopathological cohort, we identified IDH1 as a crucial target that confers chemoresistance to GEM in iCCA. Mechanistically, wtIDH1 oxidizes isocitrate to generate αKG and NADPH, which help mitigate GEM-induced oxidative stress and maintain cellular redox homeostasis. Significantly, pharmacological inhibition of wtIDH1 using GSK321 or the FDA-approved inhibitor ivosidenib demonstrated synergistic antitumor efficacy with GEM in preclinical iCCA models, particularly in the context of TME. Herein, our findings highlight the role of wtIDH1-dependent antioxidant responses in facilitating chemoresistance in iCCA, a mechanism that may also extend to other solid tumors harboring wtIDH1. The combination of the FDA-approved ivosidenib targeting IDH1 with conventional chemotherapy represents a promising treatment approach for patients with iCCA with wtIDH1, warranting further exploration and validation in clinical trials.

## Results

### Higher IDH1 expression was associated with the resistance of iCCA to GEM-based chemotherapy.

**Figure 1:**
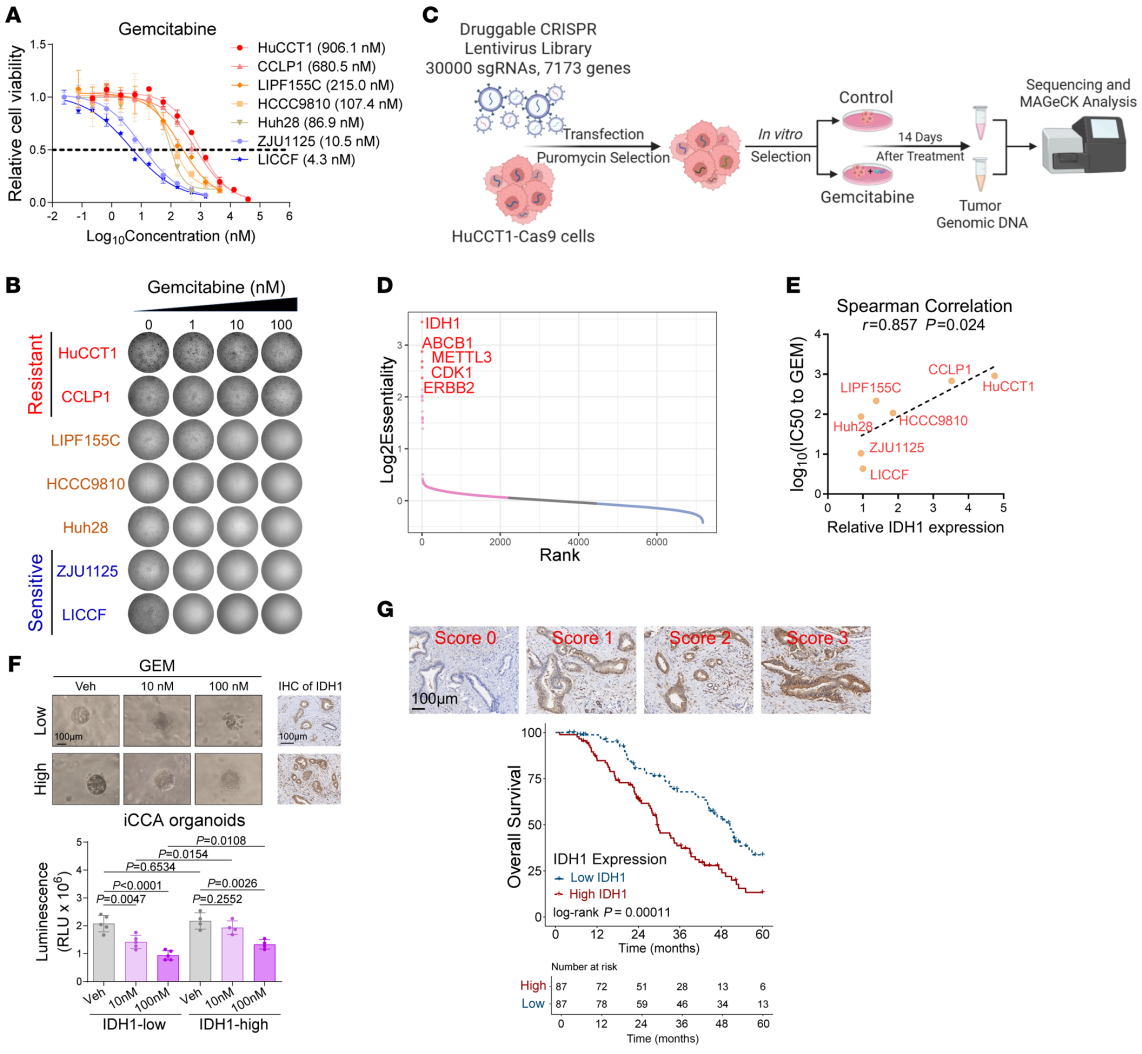
Higher IDH1 was associated with the resistance of iCCA to GEM-based chemotherapy. (**A**) Luminescence denoted relative cellular viability of iCCA cells treated with serial concentrations of GEM for 72 hours (*n* = 3 replicates), and the calculated IC_50_ are shown in brackets. (**B**) Colony-formation assay of iCCA cells treated with serial concentrations of GEM for 10 days. (**C**) Workflow of the synthetic lethal screen in GEM-resistant HuCCT1 cells with druggable CRISPR/Cas9 library screening (*n* = 3 replicates). (**D**) Dot plot showing the synthetic lethality of each gene in promoting HuCCT1 resistance to 14-day GEM treatment (1 μM), where IDH1 was identified as the top synthetic lethal hit. (**E**) Spearman’s correlation analysis of the IDH1 expression level, with IC_50_ in response to GEM in iCCA cells. (**F**) Tumor organoids derived from patients with iCCA were treated with GEM for 72 hours to evaluate their relative viability (*n* = 9). These organoids were dichotomized by the IDH1 expression level of their corresponding primary tumor sections. Scale bar: 100 μm. (**G**) Kaplan-Meier OS analysis of patients with iCCA who received radical resection and adjuvant GEM-based chemotherapy in the SYSMH cohort (*n* = 174). Representative tiles of IDH1 IHC staining are shown below, and samples with IHC score >1.3 were classified as “IDH1 high.” Scale bar: 100 μm. Statistical analysis was performed with (**E**) Spearman’s correlation coefficient, (**F**) 1-way ANOVA, and (**G**) log-rank test. Data represent mean ± SEM. See also Supplemental Figure 1.

GEM-based chemotherapy remains the most recommended treatment for patients with advanced iCCA; however, chemoresistance to GEM diminishes the therapeutic benefits (13). To address this challenge, we first assessed the sensitivity of various iCCA cell lines to GEM, identifying those with primary resistance to GEM (Figure 1, A and B). We then constructed a druggable CRISPR/Cas9 library screen (Figure 1C), as previously reported (33–35), to identify key targets associated with chemoresistance to GEM in iCCA. Sequencing results demonstrated stable read counts across samples and high intragroup correlation (Supplemental Figure 1, A and B; supplemental material available online with this article; https://doi.org/10.1172/JCI199730DS1), confirming the stability and reproducibility of our in vitro screening model. Scatter plots highlighted the importance of specific genes in facilitating chemoresistance in iCCA to GEM, with IDH1 emerging as a critical target (Figure 1D).

IDH1 is an important enzyme in the TCA cycle, catalyzing the oxidative decarboxylation of isocitrate to produce CO_2_ and αKG, which is crucial for maintaining cellular redox homeostasis (30). In fact, IDH1 mutations are the most common mutation pattern in iCCA (26–28), promoting tumorigenesis and progression via (R)-2HG–mediated epigenetic/metabolic reprogramming. However, iCCA cell lines (HuCCT1 and CCLP1) identified with primary resistance to GEM are devoid of IDH1 mutations. Thus, the effect of IDH1 on chemoresistance to GEM may be dependent on the physiological function of wtIDH1. In assessing the basal expression of IDH1 in iCCA cell lines, we observed higher IDH1 expression in the resistant cell lines (Supplemental Figure 1C), none of which harbors IDH1/IDH2 hotspot mutations (Supplemental Figure 1D), and the resistance to GEM was positively correlated with quantified IDH1 expression (Figure 1E). Moreover, resected recurrent iCCA tumors after GEM-based chemotherapy demonstrate higher IDH1 expression compared with paired iCCA primary tumors (Supplemental Figure 1E), and sensitive iCCA cell lines surviving GEM treatment showed increased IDH1 expression (Supplemental Figure 1F), suggesting that IDH1 contributes to the maintenance of survival of iCCA cells under GEM treatment. The elevated IDH1 expression in residual pancreatic ductal adenocarcinoma cells, a kind of malignancy showing a ductal phenotype similar to iCCA that is mainly treated with GEM-based regimens, after GEM treatment from GEO datasets (36, 37) provided supportive evidence (Supplemental Figure 1G).

Furthermore, using our constructed patient-derived organoids library, we verified that higher IDH1 expression in iCCA correlates with greater resistance to GEM (Figure 1F). In clinicopathological analysis of patients with iCCA, IDH1 was specifically expressed in iCCA cells (Supplemental Figure 1H) and was associated with poor OS in patients with iCCA treated with radical surgery and GEM-based chemotherapy (Figure 1G). Further univariate and multivariate Cox regression analysis identified the adverse prognostic impact of IDH1 expression regardless of IDH1 mutation status (Supplemental Figure 1I and Supplemental Table 1). These findings suggest that IDH1 expression facilitates GEM resistance in iCCA tumors.

### Inhibition of IDH1 alleviates the chemoresistance of iCCA to GEM.

**Figure 2:**
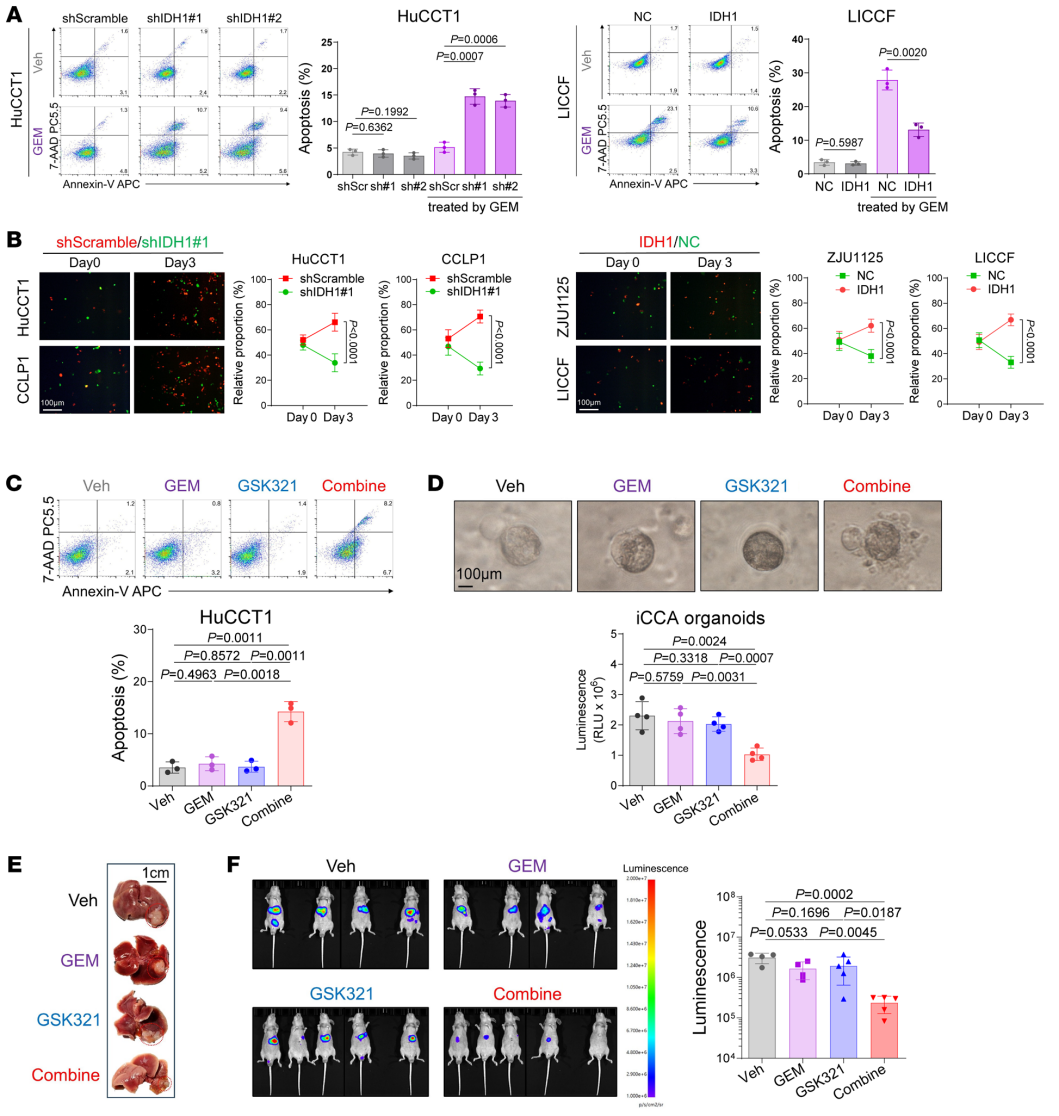
Inhibition of IDH1 alleviates the chemoresistance of iCCA to GEM. (**A**) Apoptosis flow cytometry evaluating the resistance of iCCA cells with altered IDH1 to GEM (10 nM, 72 hours; *n* = 3 replicates). (**B**) Human iCCA cells with altered IDH1 were cocultured in culture medium with GEM to evaluate their relative resistance to GEM (10 nM; *n* = 3 replicates). Scale bar: 100 μm. (**C**) Apoptosis flow cytometry evaluating the combination efficacy of GSK321 (1 μM) and GEM in HuCCT1 cells (10 nM, 72 hours; *n* = 3 replicates). (**D**) Box plot illustrating the relative viability of iCCA organoids after treatment of GSK321 (1 μM) and GEM for 72 hours (10 nM; *n* = 4). Scale bar: 100 μm. (**E**) Gross view of the tumors in the liver subcapsular inoculation models in nude mice. (**F**) Bioluminescence imaging analysis showing the photon flux of inoculated HuCCT1 xenografts (*n* = 18). Statistical analysis was performed with (**A**, **C**, **D**, and **F**) 1-way ANOVA and (**B**) 2-sided *t* test. Data represent mean ± SEM. See also Supplemental Figures 2 and 3.

The above results demonstrate that IDH1 is associated with chemoresistance of iCCA to GEM. Consequently, we performed lentivirus-mediated knockdown or overexpression experiments targeting IDH1 in iCCA cells. The results revealed no effect of IDH1 on tumor malignancies (Supplemental Figure 2, A–D), including proliferation, migration, and invasion. However, IDH1 overexpression increased the resistance of IDH1-low iCCA cell lines to GEM, while IDH1 knockdown reversed the resistance of IDH1-high iCCA cell lines to GEM (Figure 2A and Supplemental Figure 2, E and F), consistent with the clinicopathological correlation between IDH1 and GEM resistance. Coculturing iCCA cells harboring altered expression of IDH1 under GEM treatment further confirmed the role of IDH1 in facilitating resistance to GEM (Figure 2B).

Previous studies have elucidated the inhibitory effects of GSK321 on wtIDH1 (38). In our experiments, we found that GSK321 does not significantly affect tumor malignancies (Supplemental Figure 3, A–D), but it can overcome the primary resistance of iCCA to GEM (Figure 2, C and D, and Supplemental Figure 3, E and F), where the combination of GSK321 and GEM exhibited profound inhibition of iCCA proliferation. However, in the combination assays of GSK321 and GEM in iCCA cells with knockdown of IDH1 expression, their combination did not yield additional synergistic suppression (Supplemental Figure 3G). These results indicate that GSK321-mediated chemosensitizing effects to GEM are dependent on IDH1. Subsequently, we constructed orthotopic iCCA tumor models (liver subcapsular tumor inoculation models) and validated the synergistic effects of GSK321 with GEM (Figure 2, E and F). The combination treatment showed no influence on mouse weights (Supplemental Figure 3H) and led to reversed resistance to GEM, with reduced Ki67 tumor proliferation (Supplemental Figure 3I) and increased tumor apoptosis (Supplemental Figure 3J).

Although GSK321 exhibits stronger inhibitory effects on mutant IDH1, the lack of IDH1 mutations in identified iCCA cell lines (HuCCT1 and CCLP1) with primary resistance to GEM indicate that the effects of GSK321 in reversing chemoresistance are dependent on its inhibition of wtIDH1. These results demonstrate that targeting wtIDH1 may enhance iCCA sensitivity to GEM, representing a potentially viable therapeutic strategy to overcome chemoresistance.

### The effects of IDH1 in causing resistance of iCCA to GEM depends on its role in maintaining redox homeostasis.

We have demonstrated that wtIDH1 facilitates chemoresistance of iCCA to GEM. Given that IDH1 participates in TCA cycle metabolism and plays a crucial role in maintaining cellular redox homeostasis, while GEM induces cell death by inhibiting DNA synthesis and causing excessive cellular oxidative stress, we speculate that the underlying mechanism by which wtIDH1 leads to iCCA resistance to GEM is its function in promoting the capacity of iCCA cells to handle excessive cellular oxidative stress.

To elucidate the role of oxidative stress in iCCA chemoresistance, we first assessed the oxidative stress levels in iCCA cells treated with GEM. Although there was no significant difference in the proliferation and intracellular ROS levels of drug-resistant iCCA cells at moderate GEM concentration (Supplemental Figure 4, A and B), their glutathione (GSH)/GSH disulfide (GSSG) ratio and αKG contents were reduced (Supplemental Figure 4, C and D). We also evaluated the effect of GEM on replication stress and DNA damage in iCCA cells, where serial GEM induced dose-dependent elevated pCHK1(S345) and γH2AX expression, and the difference was consistent with the primary resistance to GEM (Supplemental Figure 4, E–G). These findings suggest that, even in the absence of overt changes in proliferation or ROS levels, GEM treatment induces metabolic alterations in iCCA cells.

**Figure 3:**
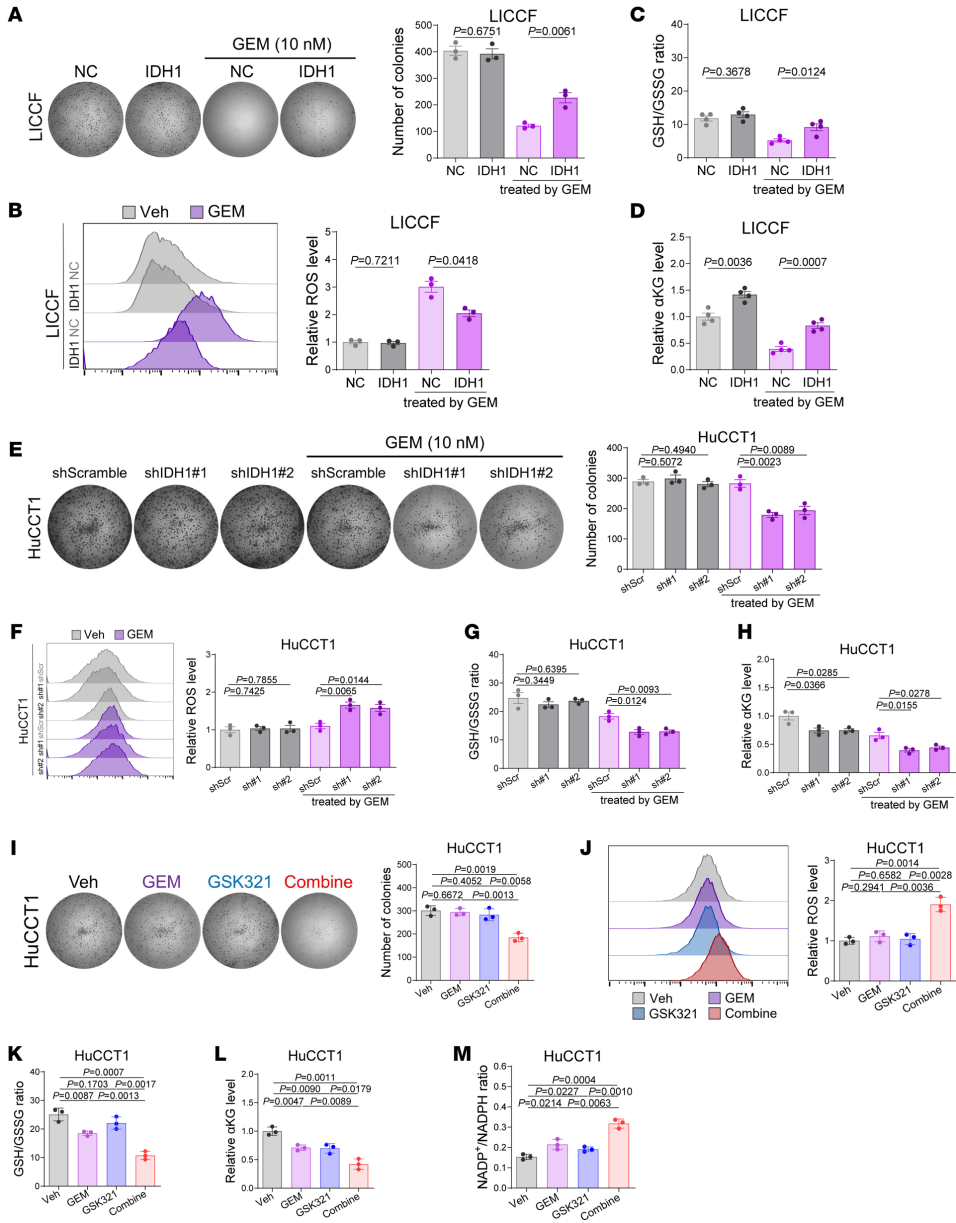
GEM treatment and IDH1 alteration affects intracellular oxidative stress in iCCA. (**A**–**D**) In vitro assays evaluating the rescue effects of overexpressing IDH1 in GEM-sensitive LICCF cells against the effect of GEM treatment (10 nM, 10 days) on the proliferation (**A**), oxidative stress (**B**), GSH/GSSG ratio (**C**), and αKG contents (**D**). (**E**–**H**) In vitro assays evaluating the synergistic effects of combining IDH1 knockdown and GEM treatment (10 nM, 10 days) in GEM-resistant HuCCT1 cells on the proliferation (**E**), oxidative stress (**F**), GSH/GSSG ratio (**G**), and αKG contents (**H**). (**I**–**M**) In vitro assays evaluating the synergistic effects of combining IDH1 pharmacological inhibition (1 μM) and GEM treatment (10 nM, 10 days) in GEM-resistant HuCCT1 cells on the proliferation (**I**), oxidative stress (**J**), GSH/GSSG ratio (**K**), αKG contents (**L**), and NADP^+^/NADPH ratio (**M**). Sample size: *n* = 3 replicates for each group. Statistical analysis was performed with 1-way ANOVA. Data represent mean ± SEM. See also Supplemental Figures 4 and 5.

Consistent with our assumption, overexpression of IDH1 did not significantly affect the proliferation or oxidative stress of iCCA cells under normal culture conditions; however, it rescued the inhibitory effects and oxidative stress responses induced by GEM treatment in GEM-sensitive iCCA cells (Figure 3, A–D, and Supplemental Figure 5, A–D). In addition, IDH1 knockdown combined with GEM treatment led to proliferation inhibition, intracellular ROS spiking, and a reduction in GSH/GSSG ratio as well as a reduction in αKG contents in GEM-resistant iCCA cells (Figure 3, E–H, and Supplemental Figure 5, E–H). These synergistic effects were also observed when combining GEM and GSK321 to inhibit wtIDH1 (Figure 3, I–M, and Supplemental Figure 5, I–M).

**Figure 4:**
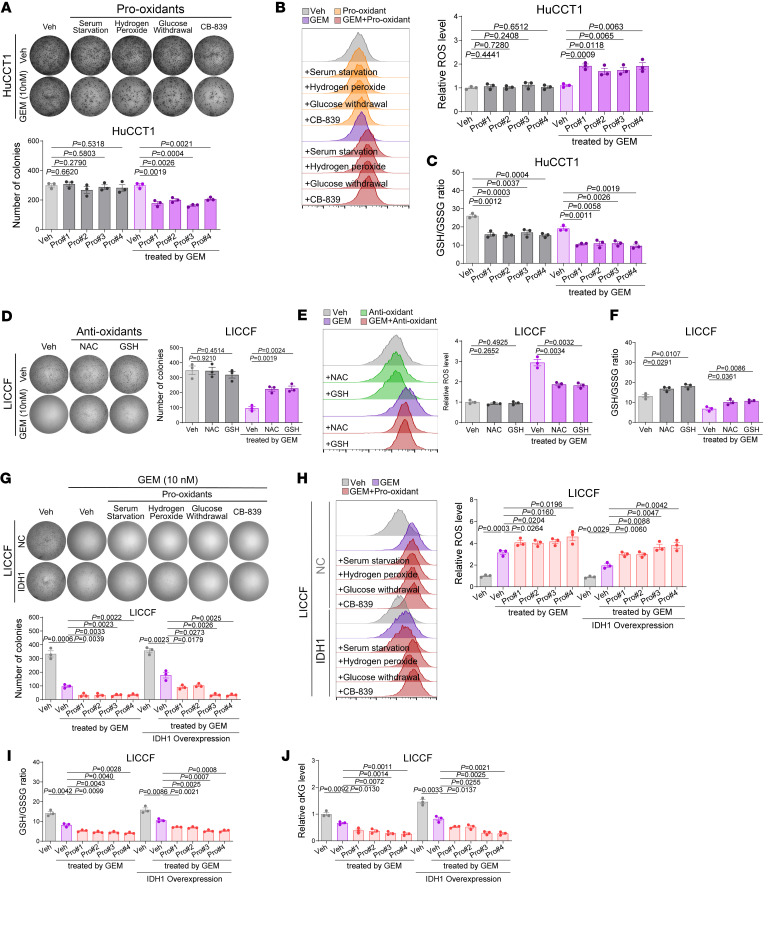
The effects of IDH1 in causing resistance of iCCA to GEM depend on its role in maintaining redox homeostasis. (**A**–**C**) In vitro assays evaluating the synergistic effects of combining prooxidants (1% serum starvation, 10 μM hydrogen peroxide, 2.5 mM glucose withdrawal, and 1 μM glutaminase inhibitor CB-839) and GEM treatment (10 nM, 10 days) in GEM-resistant HuCCT1 cells on the proliferation (**A**), oxidative stress (**B**), and GSH/GSSG ratio (**C**). (**D**–**F**) In vitro assays evaluating the rescue effects of supplementing antioxidants (1 mM N-acetylcysteine, and 4 mM glutathione) in GEM-sensitive LICCF cells against the effect of GEM treatment (10 nM, 10 days) on the proliferation (**D**), oxidative stress (**E**), and GSH/GSSG ratio (**F**). (**G**–**J**) In vitro assays evaluating the rescue effects of overexpressing IDH1 in GEM-sensitive LICCF cells against the synergistic efficacy of combining GEM treatment (10 nM, 10 days) with prooxidants (1% serum starvation, 10 μM hydrogen peroxide, 2.5 mM glucose withdrawal, and 1 μM glutaminase inhibitor CB-839) on the proliferation (**G**), oxidative stress (**H**), GSH/GSSG ratio (**I**), and αKG contents (**J**). Sample size: *n* = 3 replicates for each group. Statistical analysis was performed with (**A**–**F**) 1-way ANOVA and (**G**–**J**) 2-way ANOVA. Data represent mean ± SEM. See also Supplemental Figures 6 and 7.

**Figure 5:**
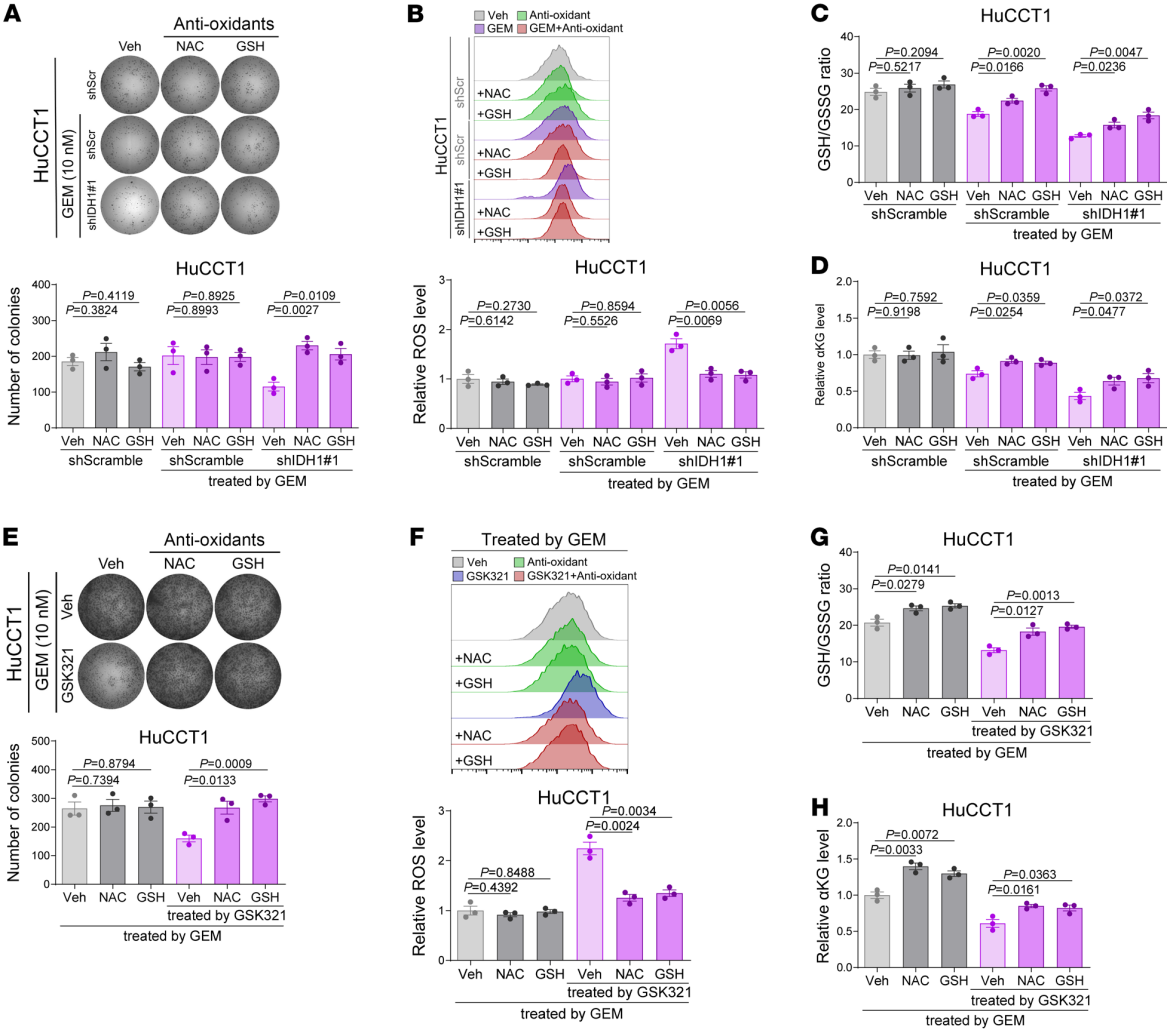
Supplementing antioxidants counteracts the synergistic efficacy of knocking down or pharmacologically inhibiting IDH1 in reversing chemoresistance. (**A**–**D**) In vitro assays evaluating the rescue effects of supplementing antioxidants (1 mM N-acetylcysteine, and 4 mM glutathione) in GEM-resistant HuCCT1 cells against the synergistic efficacy of combining IDH1 knockdown and GEM treatment (10 nM, 10 days) on proliferation (**A**), oxidative stress (**B**), GSH/GSSG ratio (**C**), and αKG contents (**D**). (**E**–**H**) In vitro assays evaluating the rescue effects of supplementing antioxidants (1 mM N-acetylcysteine and 4 mM glutathione) in GEM-resistant HuCCT1 cells against the synergistic efficacy of combining IDH1 pharmacological inhibition (1 μM) and GEM treatment (10 nM, 10 days) on proliferation (**E**), oxidative stress (**F**), GSH/GSSG ratio (**G**), and αKG contents (**H**). Sample size: *n* = 3 replicates for each group. Statistical analysis was performed with (**A**–**D**) 2-way ANOVA and (**E**–**H**) 1-way ANOVA. Data represent mean ± SEM. See also Supplemental Figures 6 and 7.

To investigate whether wtIDH1-mediated resistance of iCCA to GEM is dependent on the management of oxidative stress, we first confirmed that the additional combination of prooxidants could inhibit the growth of GEM-resistant iCCA cells under GEM treatment (Figure 4, A–C, and Supplemental Figure 6, A–C), effectively killing iCCA cells that were on the brink of excessive stress under moderate GEM treatment. In addition, supplementing antioxidants N-acetylcysteine and GSH rescued the effects of GEM, including proliferation inhibition and oxidative stress induction, in GEM-sensitive iCCA cells (Figure 4, D–F, and Supplemental Figure 6, D–F). Furthermore, treatment with prooxidants attenuated the prochemoresistance of IDH1 overexpression in GEM-sensitive iCCA cells (Figure 4, G–J, and Supplemental Figure 6, G–J), exacerbating the oxidative stress caused by GEM. Meanwhile, supplementation of antioxidants abolished the chemosensitization effects of IDH1 knockdown in GEM-resistant iCCA cells (Figure 5, A–D, and Supplemental Figure 6, K–N), contributing to intracellular redox homeostasis. The rescue effects of antioxidants were also validated in experiments involving the combination of GSK321 and GEM treatment (Figure 5, E–H, and Supplemental Figure 6, O–R).

Our results illustrate that wtIDH1 inhibition leads to reduced αKG contents, decreased GSH/GSSG ratio, and elevated NADP^+^/NADPH ratio. Since αKG is a substrate for transaminase-dependent synthesis of glutamate, a requisite component of the GSH tripeptide, we supposed that decreased de novo GSH synthesis is the major contributor to impaired redox defense upon wtIDH1 inhibition. To test this hypothesis, we transduced HuCCT1 and CCLP1 cells with mitochondrial TPNOX, an engineered enzyme that accelerates NADPH catabolism (Supplemental Figure 7, A and B) (39). The induction of mitochondrial TPNOX elevated the NADP^+^/NADPH ratio, and this elevation was further amplified when combined with GEM (Supplemental Figure 7C), consistent with their suppression effects on tumor proliferation (Supplemental Figure 7D). Conversely, additional IDH1 inhibition under TPNOX-induced conditions did not further increase the NADP^+^/NADPH ratio (Supplemental Figure 7E) or enhance the inhibition effects on tumor growth (Supplemental Figure 7F), proving that the chemosensitizing activity of IDH1 inhibition is dependent on the effect of IDH1 inhibition on NADPH catabolism. These results demonstrate that TPNOX-mediated NADPH catabolism could recapitulate the GEM-sensitizing effects of IDH1 inhibition, consistent with our hypothesis that impaired redox defense upon wtIDH1 inhibition was mainly due to decreased de novo GSH synthesis.

Taken together, these findings demonstrate that wtIDH1 promotes resistance of iCCA to cytocidal GEM by enhancing iCCA’s ability to counteract excessive oxidative stress and maintain intracellular redox homeostasis.

### Allosteric IDH1 inhibitor ivosidenib inhibits wtIDH1 to reverse the resistance of iCCA to GEM.

Currently, ivosidenib, a drug targeting mutant IDH1, is well tolerated in patients and has already been approved for the treatment of IDH1-mutant advanced iCCA patients, demonstrating promising therapeutic effects in multicenter clinical trials (5, 6). Although ivosidenib was primarily designed to target mutant IDH1, recent reports suggest that it also exhibits high binding potency and an inhibitory effect on wtIDH1 (cell-free half-maximal inhibitory concentration of 17.6 nM) within specific TMEs (31) as well as in enzymatic tests: GSK321 IC_50_ = 2.9~4.6 nM against IDH1 R132 mutations, GSK321 IC_50_ = 46 nM against wtIDH1, ivosidenib IC_50_ = 8~13 nM against IDH1 R132 mutations, ivosidenib IC_50_ = 71 nM against wtIDH1 (38, 40).

**Figure 6:**
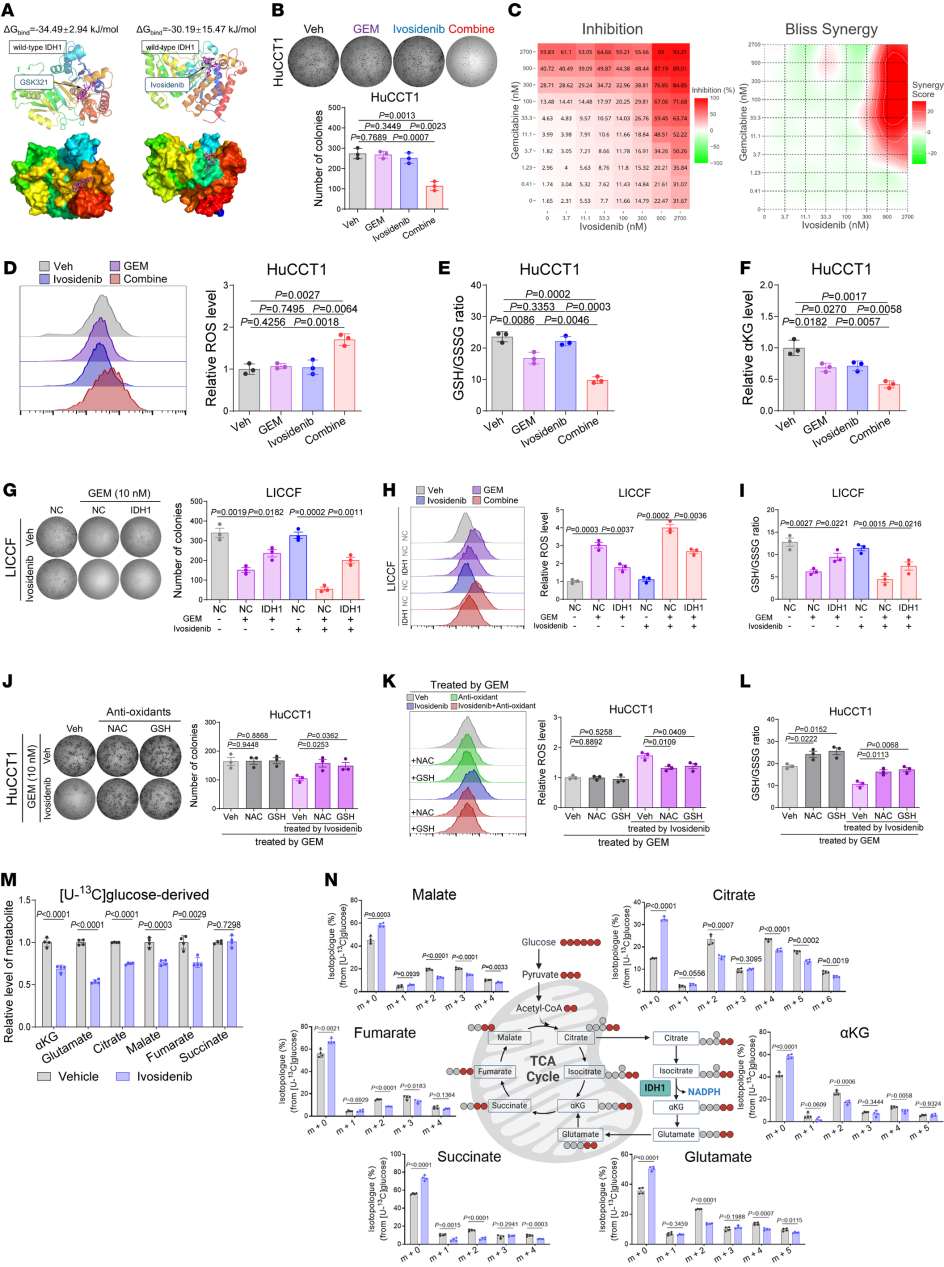
Allosteric IDH1 inhibitor ivosidenib inhibits wtIDH1 to reverse the resistance of iCCA to GEM. (**A**) Molecular docking between wtIDH1 and GSK321 or ivosidenib, where the Gibbs binding energy (ΔG_bind_) was determined by MM-PBSA algorithm (57). (**B**) Colony-formation assay of GEM-resistant HuCCT1 cells treated with GEM (10 nM) and ivosidenib (1 μM) for 10 days under magnesium-low complete medium. (**C**) Relative viability and corresponding Bliss independence scores in response to the combination of ivosidenib and GEM in GEM-resistant HuCCT1 cells for 10 days under magnesium-low complete medium, where positive values represent synergistic response. (**D**–**F**) In vitro assays evaluating the synergistic effects of combining ivosidenib (1 μM) and GEM treatment (10 nM, 10 days) in GEM-resistant HuCCT1 cells on the oxidative stress (**D**), GSH/GSSG ratio (**E**), and αKG contents (**F**). (**G**–**I**) In vitro assays evaluating the reversal effects of applying ivosidenib (1 μM) in GEM-sensitive LICCF cells for 10 days against the rescue effects of overexpressing IDH1 on proliferation (**G**), oxidative stress (**H**), and GSH/GSSG ratio (**I**). (**J**–**L**) In vitro assays evaluating the rescue effects of supplementing antioxidants (1 mM N-acetylcysteine and 4 mM glutathione) in GEM-resistant HuCCT1 cells for 10 days against the synergistic efficacy of combining ivosidenib (1 μM) and GEM treatment (10 nM) on proliferation (**J**), oxidative stress (**K**), and GSH/GSSG ratio (**L**). (**M** and **N**) Metabolic profiling demonstrating the glucose-dependent isotopologues (**M**) and isotopologue distribution (**N**) in HuCCT1 cells pretreated by ivosidenib (1 μM) or vehicle for 7 days, which were cultured under glucose-depleted medium for a 30-hour washout period followed by 6 hours of additional 2.5 mM [U-^13^C] glucose culturing under magnesium-low medium. Sample size: *n* = 3 replicates for each group, except that *n* = 4 replicates for **M** and **N**. Statistical analysis was performed with (**B**, **D**–**F**, and **J**–**L**) 1-way ANOVA, (**G**–**I**) 2-way ANOVA, and (**M** and **N**) 2-sided t test. Data represent mean ± SEM. See also Supplemental Figures 8 and 9.

In our molecular docking analyses of ivosidenib with wtIDH1, ivosidenib presents binding affinity to the catalytic domain of wtIDH1 with a Gibbs binding energy (ΔG_bind_) of –30.19 ± 15.47 kJ/mol, which is comparable to that of GSK321 binding with wtIDH1 (ΔG_bind_ = –34.49 ± 2.94 kJ/mol), suggesting the inhibitory potential of ivosidenib on wtIDH1 (Figure 6A). However, under conventional culture conditions, ivosidenib treatment alone did not affect the proliferation or oxidative stress of iCCA cells (Supplemental Figure 8, A–D) and neither did it reverse iCCA’s resistance to GEM (Supplemental Figure 8E). Previous studies have reported that magnesium (Mg^2+^) competitively interacts with Asp279 in the allosteric pocket of wtIDH1 to prevent the binding of inhibitors targeting the catalytic pocket of IDH1, thus reducing the inhibitory effect of ivosidenib of wtIDH1 (41). Therefore, we conducted combination treatment experiments under low-Mg^2+^ conditions and demonstrated the synergistic antitumor effects of ivosidenib and GEM (Bliss synergy indices >20) in iCCA cells (Figure 6, B–F, and Supplemental Figure 9, A–E).

Our previous findings indicate that wtIDH1 facilitates the resistance of iCCA to GEM by enhancing the capacity of iCCA cells to revolve GEM-induced oxidative stress. Under low-Mg^2+^ conditions, we found that the combination of ivosidenib and GEM demonstrated synergistic antitumor effects and led to redox imbalance in iCCA cells, thereby overcoming the chemoresistance conferred by overexpression of IDH1 (Figure 6, G–I, and Supplemental Figure 9, F–H). Additionally, supplementing antioxidants reversed the synergistic effects of ivosidenib and GEM in iCCA (Figure 6, J–L, and Supplemental Figure 9, I–K), highlighting the dependence of ivosidenib on redox disturbance to exert its antitumor effects. Given the proposed redox mechanism, we constructed in vivo orthotopic iCCA tumor models to evaluate the 24-hour acute effect of GEM and ivosidenib on redox imbalance. As illustrated in Supplemental Figure 9, L–O, administration of GEM alone caused acute redox imbalance, including increased lipid peroxidation (increased 4-HNE), DNA oxidation (increased 8-oxo-dG), and DNA damage (increased γH2AX and pCHK1), with decreased antioxidant capacity (decreased GSH/GSSG ratio and increased NADP^+^/NADPH ratio), whereas the combination with ivosidenib exhibited synergistic effects. These results have strengthened the mechanistic link among replication stress, DNA damage, redox imbalance, and GEM-induced cell death.

The mitochondrial dysfunction induced by the inhibition of wtIDH1 with ivosidenib was further confirmed through metabolic flux assays (Figure 6, M and N). Treatment with ivosidenib led to a reduction in the de novo synthesis of ^13^C-enriched metabolites related to TCA cycle, suggesting a decreased carbon input from [U-^13^C]glucose into mitochondrial TCA metabolism, thereby attenuating the IDH1-dependent TCA metabolic flux.

Taken together, these results demonstrate that under low-Mg^2+^ conditions, the allosteric IDH1 inhibitor ivosidenib effectively inhibits the physiological function of wtIDH1 in managing oxidative stress, thereby reversing the chemoresistance of iCCA to GEM.

### Ivosidenib possesses an inhibitory effect on wtIDH1 in iCCA under physiological TME.

**Figure 7:**
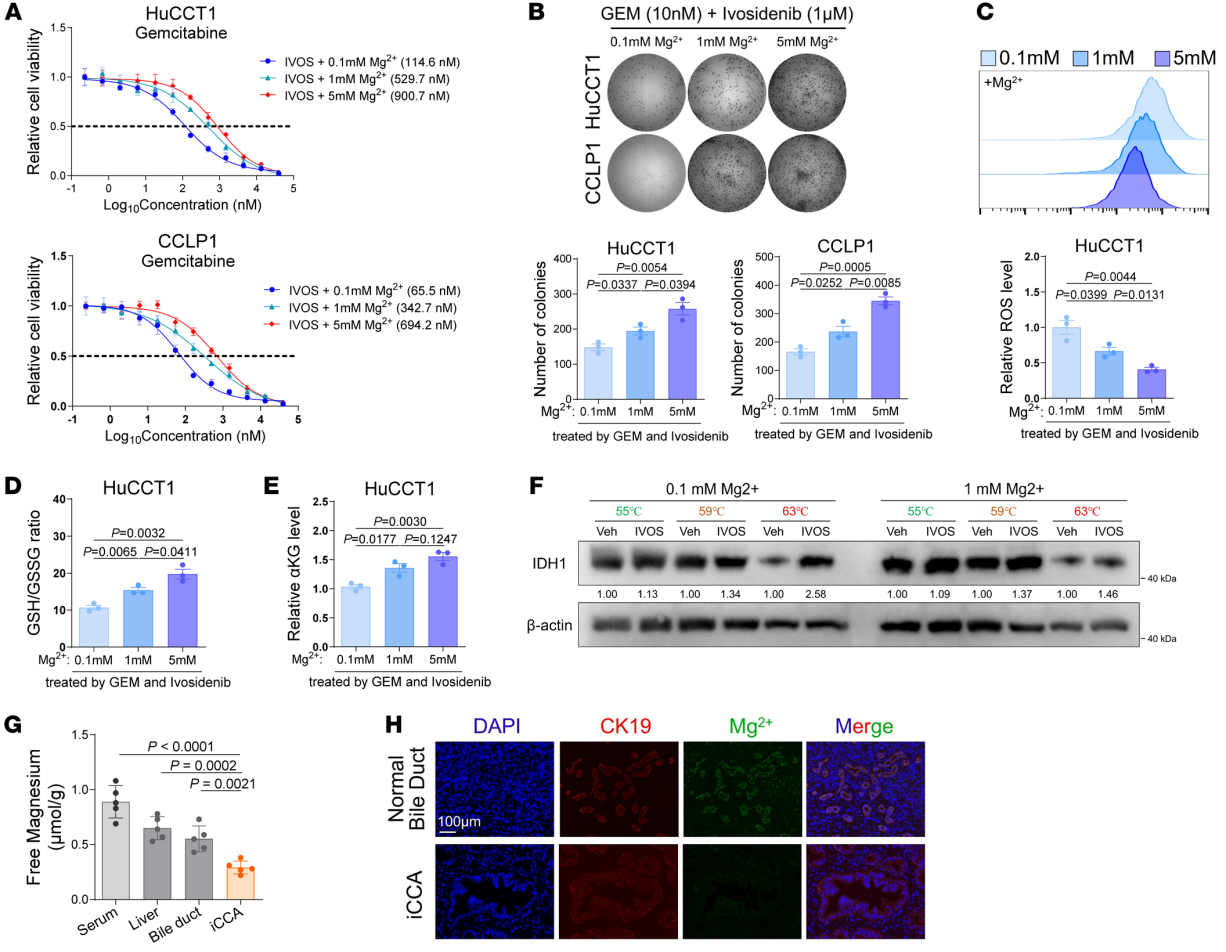
Ivosidenib possesses inhibitory effect on wtIDH1 in iCCA under physiological TME. (**A**) Luminescence-denoted relative cellular viability of iCCA cells treated with ivosidenib and GEM under serial concentrations of magnesium for 72 hours. (**B**) Colony-formation assay of iCCA cells treated with ivosidenib and GEM under serial concentrations of magnesium for 10 days. (**C**–**E**) In vitro assays evaluating the effect of serial concentrations of magnesium on the synergistic effects of combining ivosidenib (1 μM) and GEM treatment (10 nM; 10 days) in GEM-resistant HuCCT1 cells on the oxidative stress (**C**), GSH/GSSG ratio (**D**), and αKG contents (**E**). (**F**) Western blot evaluating the thermal stability of IDH1 protein in HuCCT1 cells treated by vehicle or ivosidenib (1 μM) under culture medium containing indicated concentrations of magnesium. (**G**) Spectrophotometry detecting the free magnesium levels in serum, liver, normal bile duct, and iCCA tumors collected from patients with iCCA. (**H**) Immunofluorescence staining of CK19 and fluorescent probing of magnesium in normal bile duct tissues or iCCA tumors to evaluate the distribution of magnesium. Scale bar: 100 μm. Sample size: *n* = 3 replicates for each group, except that *n* = 5 for **G**. Statistical analysis was performed with (**B**–**E**) 1-way ANOVA and (**G**) 2-sided t test. Data represent mean ± SEM. See also Supplemental Figure 10.

Our findings illustrate the therapeutic effects of ivosidenib in reversing iCCA’s resistance to GEM under low-magnesium conditions, while these effects could be neutralized by increasing Mg^2+^ levels (Figure 7, A–E, and Supplemental Figure 10, A–C). Furthermore, we observed enhanced thermal stability of intracellular wtIDH1 protein under ivosidenib treatment in low-Mg^2+^ conditions, while no stabilizing effects of ivosidenib on wtIDH1 were observed at high-Mg^2+^ levels (Figure 7F). These observations suggest that Mg^2+^ competes with ivosidenib for binding to wtIDH1, thereby relieving the inhibitory effects of ivosidenib on wtIDH1, including resistance to GEM and management of oxidative stress.

To date, no comprehensive analyses have been conducted on the Mg^2+^ contents and distribution in intrinsic tumor and normal tissues in patients with iCCA. To investigate the correspondence between the low-magnesium microenvironment and the physiological body microenvironment, we collected human samples, including peripheral blood, normal liver, normal bile duct, and cholangiocarcinoma tissues from patients with iCCA for Mg^2+^ content measurement. The results revealed high-Mg^2+^ concentration in peripheral blood and moderate-to-high-Mg^2+^ contents in normal liver and bile ducts. In contrast, Mg^2+^ levels in iCCA tumor tissues were lower due to poor blood supply (Figure 7G). The differential intracellular Mg^2+^ contents were further verified by probe-detected spatial distribution of Mg^2+^ in normal bile duct and iCCA samples (Figure 7H).

These results reveal the physiological relevance of the low-Mg^2+^ concentration used in our in vitro experiments to the intrinsic TME of iCCA. Given that Mg^2+^ can decrease the binding of ivosidenib to wtIDH1, the lower Mg^2+^ content in the iCCA microenvironment guarantees the sensitivity of iCCA expressing wtIDH1 to ivosidenib, while sparing normal tissues from its inhibitory effects. This differential sensitivity expands the therapeutic window for ivosidenib in clinical applications.

### Allosteric IDH1 inhibitor ivosidenib synergies with GEM against iCCA with wtIDH1 in preclinical models.

We previously demonstrated that, within the liver microenvironment, ivosidenib can inhibit wtIDH1 and reverse chemoresistance to GEM in iCCA. If the therapeutic efficacy of combining FDA-approved ivosidenib and GEM can be validated across various preclinical models, this therapy strategy could be rapidly translated into clinical applications to overcome chemoresistance in iCCA.

**Figure 8:**
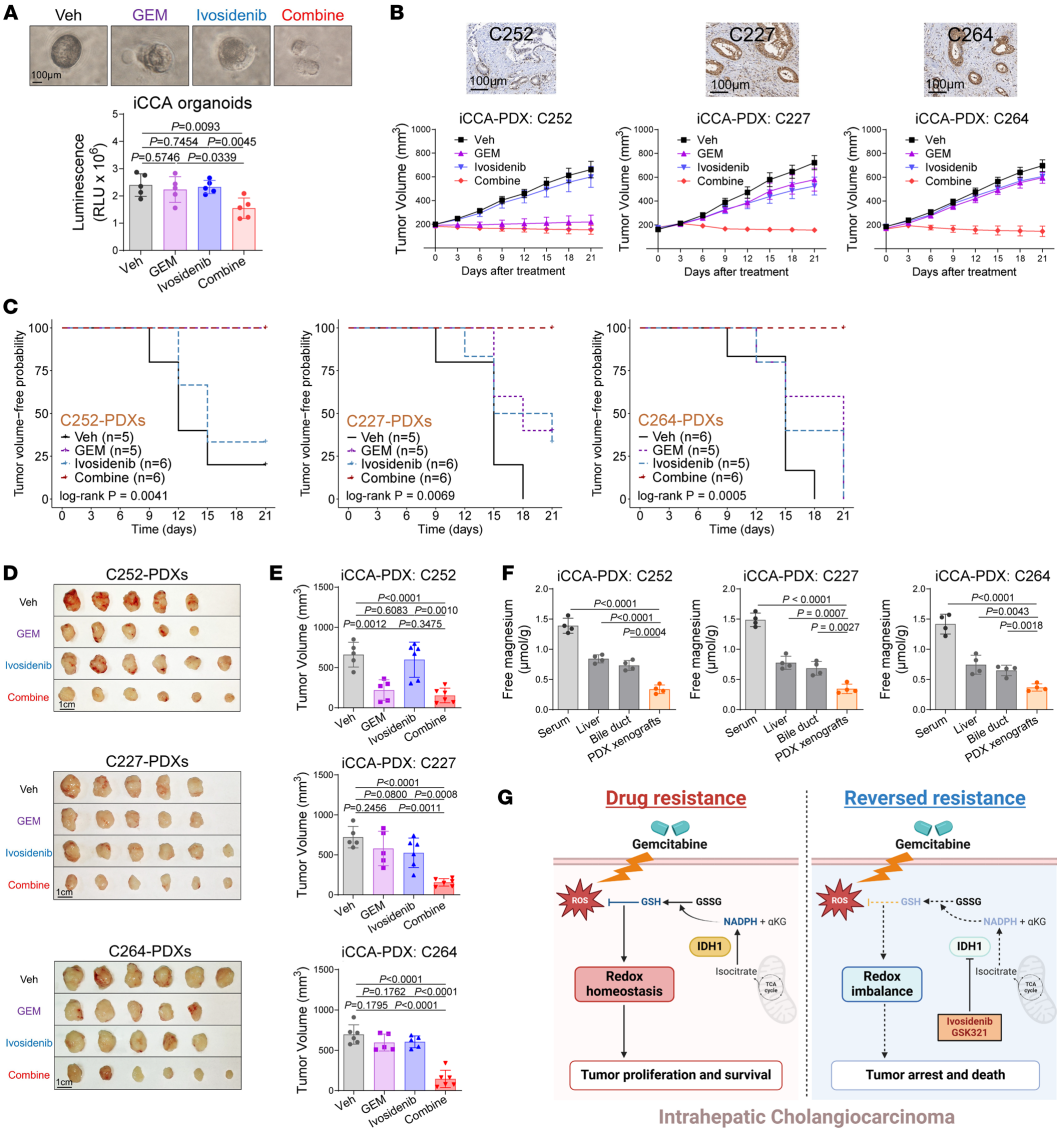
Allosteric IDH1 inhibitor ivosidenib synergies with GEM against iCCA with wtIDH1 in preclinical models. (**A**) Box plot illustrating the relative viability of iCCA organoids after treatment of ivosidenib and GEM for 72 hours (*n* = 5). Scale bar: 100 μm. (**B**) Tumor growth curves of iCCA PDX xenografts from 3 chemotherapy-naive patients with iCCA. Representative tiles of IDH1 IHC staining are shown below. Scale bar: 100 μm. (**C**) Kaplan-Meier tumor volume-free (500 mm^3^) analysis of iCCA PDX xenografts. (**D**) Gross view of the iCCA PDX xenografts after treatment of ivosidenib and/or GEM. (**E**) Box plot showing the tumor volume of iCCA PDX xenografts. (**F**) Spectrophotometry detecting the free magnesium levels in serum, liver, normal bile duct, and xenografts collected from the iCCA PDX models. (**G**) Molecular mechanisms and therapeutic vulnerability of IDH1 in iCCA’s chemoresistance to GEM. In iCCA cells, overexpressed IDH1 oxidizes isocitrate to generate αKG and NADPH, thereby reducing the oxidative stress induced by GEM, maintaining redox homeostasis, and ultimately leading to their chemoresistance to GEM. If IDH1 inhibitors (such as ivosidenib or GSK321) are used in combination with GEM, they can inhibit the catalytic function of IDH1, reducing the production of NADPH and GSH involved in antioxidant stress responses. This leads to intracellular redox imbalance, consequently reversing the chemoresistance of iCCA to GEM and resulting in tumor proliferation arrest and cell death. The image was created with BioRender. Statistical analysis was performed with (**A** and **E**) 1-way ANOVA, (**C**) log-rank test, and (**F**) 2-sided *t* test. Data represent mean ± SEM. See also Supplemental Figures 11 and 12.

To validate the synergistic antitumor effects of combining ivosidenib and GEM, we constructed several preclinical models. In the iCCA patient-derived organoids models with high IDH1 expression, ivosidenib synergized with GEM to inhibit the proliferation of iCCA organoids under low-magnesium conditions (Figure 8A).

Next, we established 3 patient-derived xenograft (PDX) models to evaluate the in vivo therapeutic efficacy of GEM and ivosidenib. Of these, C227 and C264 exhibited high IDH1 expression, while C252 showed low IDH1 expression. The IDH1-high PDXs displayed resistance to GEM, which could be overcome by the addition of ivosidenib, whereas the IDH1-low PDXs exhibited sensitivity to GEM with minimal additional effect from ivosidenib (Figure 8, B–E). Immunohistochemical staining for Ki67 proliferation (Supplemental Figure 11A) and cleaved caspase-3 apoptosis (Supplemental Figure 11B) in tumor tissues confirmed the antitumor effects of combination therapy. The reduction in the GSH/GSSG ratio (Supplemental Figure 11C) and αKG levels (Supplemental Figure 11D) in PDX tumors further verified the effect of ivosidenib and GEM on exacerbating tumor oxidative stress. Additionally, analysis of Mg^2+^ levels in peripheral blood, normal liver, normal bile duct, and iCCA PDXs tissues from tumor-bearing mice corroborated the distribution differences of Mg^2+^ across heterogeneous microenvironments (Figure 8F). These findings demonstrate that ivosidenib holds a favorable therapeutic window both in humans and mice.

Finally, we evaluated the sensitivity to GEM and effects of IDH1 inhibition in iCCA cell lines with IDH1 mutations. The RBE cells harbor IDH1 R132S hotspot mutations and demonstrated great sensitive to GEM treatment (Supplemental Figure 12, A and B), which might be attributed to the enhanced NADPH consumption by mutant IDH1, suggesting that patients with iCCA with IDH1 hotspot mutations may derive enhanced benefits from GEM-based chemotherapy. In combination assays, olutasidenib (selective for IDH1 R132H and R132C mutations) did not affect RBE proliferation, whereas GEM produced growth inhibition, and their combination showed no synergistic effects (Supplemental Figure 12C). In contrast, the nonselective inhibitors GSK321 and ivosidenib suppressed RBE proliferation and exhibited synergistic antiproliferation activity when combined with GEM (Supplemental Figure 12, D and E). These data indicate that patients with oncogenic IDH1 mutations may achieve superior responses through concurrent administration of GEM and corresponding IDH1 inhibitors. On the other hand, to validate the on-target effects of ivosidenib, we introduced the IDH1 inhibitor-resistance mutation S280F into HuCCT1 cells (Supplemental Figure 12F) (42), illustrating that IDH1 S280F variant abolished the synergistic effects of ivosidenib and GEM (Supplemental Figure 12G). We also found that ivosidenib no longer increased the thermal stability of IDH1 S280F protein (Supplemental Figure 12H), indicating a loss of ivosidenib binding after S280F mutation. These data demonstrate that the IDH1 S280F mutation confers iCCA resistance to ivosidenib and confirm the on-target mechanism of ivosidenib.

In summary, we have validated the clinical application prospects of targeting wtIDH1 to reverse chemoresistance to GEM in iCCA. Our findings demonstrate that ivosidenib, an FDA-approved allosteric inhibitor originally designed to target mutant IDH1, also shows promising therapeutic outcomes in iCCA carrying wtIDH1 within the iCCA TME.

## Discussion

IDH1 mutation leads to the intracellular accumulation of the oncogenic metabolite 2-hydroxyglutaric acid, promoting tumorigenesis and progression (43). This mutation represents a promising therapeutic target in iCCA, with a prevalence of approximately 15% (26–28). The landmark multicenter phase III randomized clinical trial ClarIDHy demonstrated the encouraging efficacy of ivosidenib, an allosteric IDH1 inhibitor, in treating patients with advanced iCCA with mutated IDH1, extending the median OS from 5.1 months in the placebo group to 10.3 months in the ivosidenib group (5, 6). Despite its clinical potency, the low IDH1 mutation rate in iCCA tumor hinders the broader application of ivosidenib. Conversely, emerging studies have elucidated the key roles of wild-type metabolic enzymes in cancer progression. Metallo et al. demonstrated that wtIDH1 catalyzes αKG to isocitrate to support de novo lipogenesis in melanoma cells under hypoxic conditions, whereas suppressing wtIDH1 inhibited tumor growth (44). The dependence on reductive carboxylation by wtIDH1 has also been demonstrated for lung cancer spheroid growth (30). Calvert et al. revealed that the oxidative decarboxylation of isocitrate to produce αKG by wtIDH1 is essential for antioxidant defense in brain tumors, underscoring the dual roles of wtIDH1 in both anabolic and catabolic pathways (45). Given the essential role of wtIDH1 in maintaining intracellular redox homeostasis, several studies have utilized wtIDH1 inhibitors and validated the therapeutic promise of wtIDH1 inhibition in preclinical cancer models, displaying great antitumor effects in vitro and in vivo (31, 46, 47). Therefore, exploring the role and potential mechanisms of wtIDH1 in chemoresistance in iCCA contributes to identifying effective therapeutic strategies to overcome chemoresistance and expanding the potential beneficiary population.

GEM is a cytosine nucleoside analog that acts as a false nucleoside to be incorporated into DNA, causing the interruption of DNA replication and cell death. Owing to its effectiveness and controllable adverse reactions, GEM has been approved for first-line treatment in various solid cancers (48, 49). However, more than 60% of patients with iCCA exhibited poor therapeutic responses to GEM in the initial treatment courses, highlighting the urgency of developing comprehensive strategies to overcome chemoresistance and improve efficacy and prognosis. Previous studies have reported that excessively activated NF-κB and TGF-β signaling contribute to chemoresistance in iCCA through reduction of the ubiquitination and stabilizing oncogenic STMN1/TGFB1/G3BP1 (14, 15). Genetic alterations are also reported to be associated with chemoresistance in iCCA, where PTEN deficiency modulates the enzymatic activity of PP2Ac and further dephosphorylates DCK, a kinase crucial for generating pharmacologically active derivates of GEM to inhibit DNA replication, thus facilitating GEM efficacy (16, 17). Besides, owing to the survival stress induced by chemotherapy, tumor cells tend to undergo self-protective responses such as upregulating Bcl-2 family, promoting GPX4 transcription and stabilization, and downregulating NRXN3/caspase-3/GSDME activation to defend against apoptosis, ferroptosis, and pyroptosis (18–20). In this study, we found that high expression of wtIDH1, an important enzyme in the TCA cycle that is crucial for maintaining cellular redox homeostasis, was associated with diminished response to GEM-based chemotherapy in the iCCA cohort. Further experiments demonstrated that wtIDH1 confers chemoresistance of iCCA to GEM both in vitro and in vivo. Notably, supplementing antioxidants abolished the synergistic effects of wtIDH1 inhibition, while overexpression of IDH1 rescued the effects of additional administration of prooxidants, including serum starvation and hydrogen peroxide but not glucose withdrawal or glutaminase inhibition. These results demonstrate the dependency of iCCA chemoresistance on the reductive capacity of wtIDH1. Collectively, these findings shed light on approaches to reverse GEM chemoresistance by targeting wtIDH1 in iCCA and to identify patients with potential therapeutic benefits from GE M treatment (Figure 8G).

Our present findings, together with previous reports (30, 44, 45), have demonstrated that wtIDH1 plays a crucial role in maintaining redox homeostasis and mitochondrial function in cancer cells. GEM is a strong prooxidant, and tumors often undergo high oxidative stress due to rapid proliferation and a nutrient-limited microenvironment. Thus, leveraging these facts, the combination of wtIDH1 inhibition and GEM in treating iCCA appears to be a logical strategy. In fact, several studies have illustrated the synergistic antitumor effects of combining chemotherapy and prooxidation treatments in various cancers. Daniel et al. demonstrated that selective targeting on leukemia stem cells by applying electron transport chain complex II inhibition to overcome chemoresistance resulted in durable remissions in patients with acute myeloid leukemia (50). Zhe et al. found that treating breast cancer cells with the NPC1L1 inhibitor ezetimibe enhanced the therapeutic effect of conventional chemotherapy by inducing excessive oxidative stress (51). Nanoparticles incorporating responsive prodrugs of copper peroxides, hydrogen peroxide, and Chlorin e6 were also proved capable of amplifying oxidative stress and enabling synergistic chemotherapy in breast cancer and thyroid cancer (52, 53). In this study, we tested the efficacy and safety of combining wtIDH1 inhibition and chemotherapy in treating iCCA. As revealed in the cell line experiments, GEM alone did not affect the proliferation of GEM-resistant iCCA cells but resulted in increased intracellular oxidation stress. Coadministration of wtIDH1 inhibition using ivosidenib or GSK321 with GEM induced tumor-reducing effects in iCCA cell lines, organoid models, orthotopic mouse experiments, and PDX, with no significant systemic toxicity observed. Most importantly, our findings support the administration of FDA-approved ivosidenib to overcome chemoresistance in iCCA without IDH1 mutations. Mg^2+^ is a free ion that competitively binds to the catalytic pocket of wtIDH1 to prevent the approach of inhibitor (41). The hypovascular nature of the iCCA microenvironment leads to reduced Mg^2+^ levels in iCCA tumors and higher Mg^2+^ content in normal tissues. The differential distribution of Mg^2+^ not only makes wtIDH1 vulnerable to allosteric inhibition by ivosidenib or GSK321 in vitro and in vivo in iCCA tumors, but it also extends the therapeutic window of wtIDH1 inhibitors without causing significant toxicity to normal tissues. Additionally, the clinical application of FDA-approved ivosidenib in patients with advanced iCCA has exhibited a favorable safety profile (5, 6), supporting the practicality of its clinical administration. Moreover, our work illustrates the correlation between high expression of IDH1 and poor response to GEM in iCCA in cancer cell lines, preclinical models, and a clinicopathological cohort, suggesting that IDH1 expression is a predictive biomarker for GEM efficacy in the clinical scenario.

In this study, we demonstrate that wtIDH1 confers iCCA chemoresistance to GEM by oxidizing isocitrate to generate αKG and NADPH to cope with oxidative stress induced by chemotherapy. Furthermore, we take the practically important step to reveal that ivosidenib, an allosteric IDH1 inhibitor originally designed to target mutant IDH1 and currently in clinical application, can potentiate the antitumor effects of GEM by inducing a 2-hit effect. In addition to GEM, other commonly used chemotherapy drugs, including cisplatin and oxaliplatin, are also strong prooxidants, rendering iCCA cells especially vulnerable to additional prooxidation treatment such as wtIDH1 inhibition. These findings suggest that ivosidenib is a promising treatment option for overcoming chemoresistance in iCCA, warranting validation in prospective clinical trials. Consistent with this idea, Mehrdad et al. designed and conducted a phase Ib clinical trial (NCT05209074) to evaluate the synergistic efficacy of ivosidenib and FOLFIRINOX chemotherapy in patients with resectable pancreatic adenocarcinoma, based on their previous findings that wtIDH1 inhibition could enhance chemotherapeutic efficacy in pancreatic adenocarcinoma (47).

Our study has several limitations. First, although the combination of ivosidenib targeting wtIDH1 and GEM exhibits synergistic antitumor effects in iCCA preclinical models, its efficacy and safety in patients with iCCA remain to be explored. Further clinical trials are warranted to verify its therapeutic potential in humans. Second, the current study focused solely on the mechanisms and potential therapeutic approaches for chemoresistance to GEM in iCCA. Although other commonly used chemotherapy drugs, such as cisplatin and oxaliplatin, are also strong prooxidants and may exhibit synergistic efficacy with wtIDH1 inhibition, their actual effects and underlying mechanisms require further investigation. Finally, our study primarily examined the intrinsic molecular alterations in iCCA cancer cell that promote chemoresistance, whereas the alterations in the tumor stroma/immune microenvironment may also play crucial roles in chemoresistance and warrant further investigation. Therefore, in subsequent studies, we plan to comprehensively explore the microenvironmental alterations that confer chemoresistance to iCCA using single-cell RNA sequencing and spatial transcriptome analysis.

Collectively, these data reveal that the strong prooxidant GEM enforces the reliance of iCCA cells on wtIDH1 to maintain cellular redox homeostasis. In addition, owing to iCCA’s vulnerability to wtIDH1 inhibition, targeting wtIDH1 using ivosidenib combined with GEM in the context of iCCA TME induced a 2-hit effect and profound antitumor efficacy in vitro and in vivo. Thus, our findings provide a promising therapeutic strategy for targeting wtIDH1 to overcome chemoresistance in iCCA, warranting further clinical investigation into the safety and efficacy of ivosidenib and chemotherapy combination treatment in iCCA.

## Methods

For further details regarding the materials and methods used, please refer to the Supplemental Methods.

### Sex as a biological variable.

Sex was not considered as a biological variable for patient samples. Since no evidence shows sex differences in chemoresistance of cholangiocarcinoma, our study did not include a comparison between sexes and exclusively examined male mice.

### Study design.

The experiments in the study were designed to investigate the mechanisms underlying chemoresistance of iCCA to GEM and identify the potential therapeutic strategy. Human iCCA cancer cell lines were used to explore the functional role of wtIDH1 in facilitating iCCA chemoresistance to GEM. A range of in vitro experiments was performed in the iCCA cells, including druggable CRISPR/Cas9 library screening, drug sensitivity assays, colony formation, and ROS detection. iCCA samples were collected from consecutive patients with iCCA who underwent radical resection and lymphadenectomy and received GEM-based adjuvant chemotherapy at Sun Yat-Sen Memorial Hospital (SYSMH) of Sun Yat-Sen University between January 2017 and June 2023. All diagnoses were verified by histological examination by certified pathologists. Ethical approval was obtained from the institutional review board of SYSMH (no. SYSKY-2023-950-01), and all participants signed an informed consent form. We investigated the in vivo role of IDH1-regulated redox homeostasis in facilitating the chemoresistance of iCCA to GEM as well as therapeutic vulnerability by performing an orthotopic inoculation of human GEM-resistant iCCA cancer cells in BALB/c nude mice or constructing 3 iCCA PDX models in severe immuno-deficient NCG mice. Animal experiments conformed to the Animal Research: Reporting of In Vivo Experiments (ARRIVE) guidelines and were approved by the Institutional Animal Care and Use Committee of South China University of Technology (no. 2023071). In each experiment, mice with similar age and size were randomly assigned to each group, and the group allocation was blinded to the investigators until the treatment, data collection, and data analysis were done. No data were excluded from analysis. Sample size was determined according to previous reports (54, 55) and experimental experience, and the details of study design are given in the figures, figure legends, and supplemental materials.

### Orthotopic iCCA tumor mice model.

In orthotopic iCCA tumor mice models (liver subcapsular tumor inoculation models), 1 × 10^6^ iCCA cells were resuspended in a saline and Matrigel mixture (1:1) in a total volume of 25 μL and then orthotopically inoculated into the liver subcapsular space of 4-week male nude mice. Tumors were first allowed to grow for approximately 3 weeks, and then the mice were randomly grouped and treated with different drugs (vehicle, GEM, GSK321, or their combination). The drug therapy was performed for 3 weeks: GEM was intraperitoneally injected at a dose of 10 mg/kg every 2 days, GSK321 was intraperitoneally administered at 50 mg/kg every day, and the combination was administered with the same dose. Before completion of the treatment, the proliferation of orthotopic tumors was determined using IVIS bioluminescence imaging. Then, the mice were euthanized and the tumors were harvested for paraffin-embedding and immunochemistry staining.

### PDX model.

The PDX models of iCCA tumors were established in severe immunodeficient NCG mice to evaluate the therapeutic efficacy of GEM and ivosidenib in human iCCA tumors. Briefly, the freshly resected tumors from patients with iCCA were cut into blocks of approximately 5 mm in diameter and then subcutaneously implanted into the NCG mice (P1 generation). When reaching a volume of about 500 mm^3^, the P1 tumors were excised, sectioned, and subcutaneously implanted into naive NCG mice (P2 generation). When tumor volume reached about 100 mm^3^, the P2 mice were randomly grouped and treated with different drugs (vehicle, GEM, ivosidenib, or their combination). The drug therapy was performed for 3 weeks: GEM was intraperitoneally injected at a dose of 10 mg/kg every 2 days, ivosidenib was intraperitoneally administered at 50 mg/kg every day, and the combination was administered with the same dose. After completion of the treatment, the mice were euthanized, and the tumors were harvested for volume measurement (volume = length × width^2^/2) and paraffin-embedded for immunochemistry staining.

### CRISPR/Cas9 library screening.

To investigate the potential crucial targets that facilitate resistance of iCCA to GEM treatment, we constructed a CRISPR/Cas9 library containing 7,173 druggable genes at a coverage of 4 gRNAs per gene as well as 1,308 nontargeting control gRNAs as previously described (33–35), which was designed following the instructions of GPP sgRNA Designer of the BROAD Institute (https://portals.broadinstitute.org/gppx/crispick/public).

HuCCT1, the iCCA cell line with primary resistance to GEM, was transduced with lentiCas9-Blast (Addgene: 52962) lentivirus and selected with blasticidin for 5–7 days to screen for stable Cas9-expressing cell line, which was confirmed by anti-Flag Western blot. Then, the pooled CRISPR guide lentivirus was titrated in HuCCT1-Cas9 cells to achieve an MOI of 0.3 and was selected with 2–4 μg/mL puromycin for 5–7 days. These selected cells, in independent 15 cm plates with 70%–80% confluence to ensure at least fifteen million cells for 500X convergence of the sgRNA pool, were randomly assigned to be treated by vehicle or GEM (1 μM). During the CRISPR/Cas9 library screen, the HuCCT1 cells were treated in vitro with GEM for 14 days, then the genomic DNA of the remaining HuCCT1 cells was extracted using the QIAamp DNA Mini Kit (51304, QIAGEN). The amplicon sequencing data of gDNA from CRISPR/Cas9 library screening samples was analyzed with the MAGeCK algorithm following the instructions (56), deriving the essentiality of each gene in promoting resistance of HuCCT1 to GEM treatment.

### Statistics.

All data are reported as mean ± SD. Most of the statistical analyses were performed with R software, version 4.2.3 (https://www.r-project.org/). Clinicopathological characteristics of patients with iCCA or iCCA tumors were compared using χ^2^ tests or Fisher’s exact test for categorical variables, unpaired 2-tailed *t* test, or 1-way ANOVA for continuous variables and Spearman’s correlation for continuous variables versus continuous variables. Kaplan-Meier estimated survival analysis for OS, and the log-rank test was applied to investigate the prognostic association of specific features. Drug combination efficacy was estimated by the Bliss independence model. For all the in vitro and in vivo experiments, at least 3 biological replicates were performed under the same conditions. All statistical tests were 2 tailed. A *P* value of less than 0.05 was considered significant.

### Study approval.

Ethical approval involving clinical iCCA samples was obtained from the institutional review board of SYSMH (no. SYSKY-2023-950-01), and written informed consent was obtained from all participants. The Institutional Animal Care and Use Committee of South China University of Technology approved all experimental procedures with animals (no. 2023071), which were consistent, and experiments conformed with the ARRIVE guidelines.

### Data availability.

All data associated with this study are present in the paper, Supporting Data Values file, or the supplemental materials. The CRISPR amplicon sequencing data have been deposited to the National Genomics Data Center (GSA human database: HRA011233; https://ngdc.cncb.ac.cn/gsa-human/). Two public datasets, GSE105083 and GSE118197, were obtained from the GEO database.

## Author contributions

XL, ZS, S Lin, and ML carried out the majority of the experiments, analyzed and interpreted the data, and contributed equally to the paper. XL and ZS performed in vitro experiments. XL, S Liu, and S Lin generated patient-derived organoids and carried out orthotopic animal experiments and PDX. ZS, ML, S Liu, and FZ assisted with the animal experiments. YL and FZ provided clinical samples and technical advice. HZ, CL, and LX designed and conceived the study, provided the funding, and wrote and revised the manuscript. All authors have read and approved the manuscript. The order of the co–first authors was determined by their efforts and contributions to the manuscript.

## Conflict of interest

The authors have declared that no conflict of interest exists.

## Funding support

- National Natural Science Foundation of China (82473081 to HZ, 82472903 and 82173195 to CL, and 82173229 to LX).
- China Postdoctoral Science Foundation (2024M753783 and 2025T180659 to HZ).
- Guangzhou Key Laboratory of Precise Diagnosis and Treatment of Biliary Tract Cancer (202201020375 to CL).
- Science and Technology Program of Guangzhou (2023B03J1385 to CL and 2023A03J0700 to LX).

## Supplementary Material

Supplemental data

Unedited blot and gel images

Supporting data values

## Version 1 03/17/2026

In-Press Preview

## Version 2 05/15/2026

Electronic publication
