# Supplementary material for: The allosteric IDH1 inhibitor ivosidenib overcomes chemoresistance in intrahepatic cholangiocarcinoma models expressing wild-type IDH1
Source: J Clin Invest. 2026 Mar 17;136(10):e199730. doi: 10.1172/JCI199730 (PMC13178645; doi:10.1172/JCI199730)
Supplement: Supplemental data [file jci-136-199730-s215.pdf]

Title: The allosteric IDH1 inhibitor ivosidenib overcomes chemoresistance in intrahepatic cholangiocarcinoma models expressing wild-type IDH1

Table of contents:

Supplemental Materials and Methods..2

Supplemental Figure Legend..22

Supplemental references..37

Supplemental Materials and Methods

Sex as a biological variable

Sex was not considered as a biological variable for patient samples. Since no evidence shows sex differences in chemoresistance of cholangiocarcinoma, our study did not include a comparison between sexes and thus exclusively examined male mice.

Study design

The experiments in the study were designed to investigate the mechanisms underlying chemoresistance of iCCA to GEM and identify the potential therapeutic strategy. Human iCCA cancer cell lines were used to explore the functional role of wtIDH1 in facilitating iCCA chemoresistance to GEM. A range of in vitro experiments were performed in the iCCA cells, including druggable CRISPR/Cas9 library screening, drug sensitivity assays, colony formation, and ROS detection. The iCCA samples were collected from consecutive iCCA patients who underwent radical resection and lymphadenectomy and received GEM-based adjuvant chemotherapy at Sun Yat-Sen Memorial Hospital (SYSMH) of Sun Yat-Sen University between January 2017 and June 2023. All diagnoses were verified by histological examination by certified pathologists. Ethical approval was obtained from the institutional review board of SYSMH (SYSKY-2023-950-01), and all participants were well informed and signed an informed consent form. We investigated the in vivo role of IDH1-regulated redox homeostasis in facilitating the chemoresistance of iCCA to GEM as

well as the therapeutic vulnerability by performing an orthotopic inoculation of human GEM-resistant iCCA cancer cells in BALB/C nude mice or constructing three iCCA patient-derived xenografts models in severe immuno-deficient NCG mice. Animal experiments conform to the Animal Research: Reporting of In Vivo Experiments (ARRIVE) guidelines and were approved by the Institutional Animal Care and Use Committee of South China University of Technology (2023071). In each experiment, mice with similar age and size were randomly assigned to each group, and the group allocation was blinded to the investigators until the treatment, data collection, and data analysis were done. No data were excluded from analysis. The sample size was determined according to previous reports and experimental experience, and the details of study design are given in the corresponding figures, figure legends, and supplemental materials.

Data and materials availability: All data associated with this study are present in the paper, Supporting data values file, or the Supplemental Materials. The data of CRISPR amplicon sequencing has been deposited to the National Genomics Data Center (GSA-Human database: HRA011233; https://ngdc.cncb.ac.cn/gsa-human/). Two public datasets GSE105083 and GSE118197 were obtained from GEO database

Cell lines

The human iCCA cancer cell lines HuCCT1, HCCC9810, Huh28, LICCF, ZJU1125, LIPF155C, CCLP1, and RBE were kept by Prof. Chao Liu's lab, which were authorized

by short tandem repeat DNA profiling. All cells were verified for negative mycoplasma according to MycoAlert (Lonza, Basel, Switzerland) before using. All cells were grown at 37°C in a humidified chamber with 5% CO_2. For standard cell culture, Huh28, CCLP1, and RBE were cultured in DMEM high-glucose medium with 10% FBS, 100U/ml penicillin and streptomycin, and HuCCT1, HCCC9810, LICCF, ZJU1125, and LIPF155C were cultured in RPMI 1640 medium with 10% FBS, 100U/ml penicillin and streptomycin. For cell culturing with low magnesium (Mg^{2+}), we chose customized magnesium sulfate-depleted DMEM (Cat#DM150120, Procell) as the basic culture medium and supplemented indicated amount of $MgSO_4$.

The IDH1/IDH2 mutation status at of iCCA cells or iCCA tumors were detected using TA-cloning. Briefly, RNA was extracted from the iCCA cells or iCCA tumors and reverse transcribed to obtain cDNA. Then, IDH1 containing the R132 coding region was amplified (forward: 5'-AAGGATGCTGCAGAAGCTATAAAGAAGCATAATGTTGGCGTCA-3', and reverse: 5'-TTATTGTTATCAAGCTTTGCTCTGTGGGCTAACCCTCTGGTCC-3'), and the IDH2 containing the R140 and R174 coding regions was amplified (forward: 5'-TCAAGGTGGCGAAGCCCGTGGTGGAGATGGATGGTGATGAGAT-3' and reverse: 5'-CACCACTGCCATCTTTTGGGGTGAAGACCATTTTGAAAGTGCC-3'). The ~1kb PCR products were subcloned into a TA-cloning plasmid (Cat#6011, TAKARA), followed by bacterial transformation, monoclonal bacterial colony selection, plasmid isolation, and Sanger sequencing using M13 forward primer (5'-TGTAAAACGACGGCCAGT-3').

CRISPR/Cas9 library screening

To investigate the potential crucial targets that facilitate resistance of iCCA to GEM treatment, we constructed a CRISPR/Cas9 library containing 7173 druggable genes at a coverage of four gRNAs per gene as well as 1308 non-targeting control gRNAs as previously described (1-3), which was designed following the instructions of GPP sgRNA Designer of BROAD Institute (portals.broadinstitute.org/gppx/crispick/public).

HuCCT1, the iCCA cell line with primary resistance to GEM, was transduced with lentiCas9-Blast (Addgene: 52962) lentivirus and selected with blasticidin for 5-7 d to screen for stable Cas9-expressing cell line, which was confirmed by anti-Flag western blot. Then the pooled CRISPR guide lentivirus was titrated in HuCCT1-Cas9 cells to achieve an MOI of 0.3, and was selected with 2-4 μg/mL puromycin for 5-7 d. These selected cells, in independent 15-cm plates with 70%-80% confluence to ensure at least fifteen million cells for 500X convergence of the sgRNA pool, were randomly assigned to be treated by vehicle or GEM (1μM). After the in vitro GEM treatment CRISPR/Cas9 library screening for 14 days, the genomic DNA of the remaining HuCCT1 cells was extracted using QIAamp DNA Mini Kit (Cat#51304, QIAGEN). The amplicon sequencing data of gDNA from CRISPR/Cas9 library screening samples was analyzed with MAGeCK algorithm following the instructions, deriving the essentiality of each gene in promoting resistance of HuCCT1 to GEM treatment.

Patient-derived Organoids

Freshly-resected tumors from iCCA patients were washed in cold PBS and minced in serum-free DMEM medium to pieces smaller than 1 mm^3, which were then placed in serum-free DMEM medium containing collagenase II and DNase II at 37°C for 40-60 minutes in gentleMACS dissociator (Miltenyi Biotec). After digestion terminated by DMEM medium containing 10% FBS, the dissociated cells were filtrated through 70 μm strainer and then spun down at 300 g for 8 min. The precipitated cells were resuspended in 2-5 mL of red blood cell lysis buffer for 5-8 min to remove red blood cells and then washed with PBS. Following centrifugation at 300 g for 8 min, the precipitated cells were suspended to single cells using matrigel (1×10^5 cells in 50 μL matrigel; Cat#354234, Corning), which were plated in preheated 24-well plates and placed in 37°C incubator for solidification for 15 min. Then each well was supplemented with 500 μL of cholangiocarcinoma organoid medium containing specific components as we have previously reported, including the basic Advanced DMEM/F-12 medium and supplemented B-27, Primocin, Wnt3a, Nicotinamide, N-acetylcysteine, R-spondin-1, Noggin, hEGF, hFGF10, hGastrin I, A 83-01, Y-27632, HEPES, GlutaMAX. The viability of cholangiocarcinoma organoids was determined using CellTiter-Glo 3D Cell Viability Assay (Cat#G9682, Promega).

Plasmids Transfection

Plasmids and lentivirus for transfection were synthesized by Suzhou GenePharma Co., Ltd, China. Stable IDH1-shRNA cell lines were generated by lentiviral transduction and selected by puromycin at 2-4 μg/mL. Briefly, pGag/Pol, pVSV-G and pGFP-C-shLenti-shIDH1 or scrambled control were transfected into HEK 293T cells using FuGENE HD transfection reagent (Cat#E2311, Promega). The released lentiviruses in the supernatants of 293T cells were concentrated and mixed with polybrene before infecting tumor cells. The infected tumor cells were incubated at 37°C for 8-12 h, then the cell culture medium was removed and replaced by fresh complete medium with puromycin at 2-4 μg/mL concentration for selection for 5-7 d. The following shRNAs for IDH1 from GenePharma were used: scrambled shRNA, 5' TTCTCCGAACGTGTCACGT 3'; IDH1-shRNA#1, 5' GGAGATGAAATGACACGAATC 3'; IDH1-shRNA#2, 5' GCGTCAAATGTGCCACTATCA 3'; IDH1-shRNA#3, 5' GCAGTACAAGTCCCAGTTTGA 3'. For transfecting overexpressing cells, the vector was pLenti-CMV-mCherry-Puro-IDH1.

The generation of Tet3G-mitoTPNOX iCCA cell lines were conducted according to previous reports (4). Briefly, the pLVX-Tet3G-blasticidin vector was lentiviral-transfected into iCCA cells and selected by blasticidin, then the Tet3G cells were infected with lentivirus containing pLVX-TRE3G-mitoTPNOX vector to generate the Tet3G-mitoTPNOX iCCA cell. The expression of mitochondrial TPNOX in HuCCT1 and CCLP1

cells was induced using doxycycline (Dox, 300ng/ml) for 24 h, and the protein expression was detected through western blotting for Flag tag.

The introduction of the IDH1 inhibitor-resistance mutation (IDH1 S280F mutation) in HuCCT1 cells was performed using the CRISPR-Cas9 ribonucleoprotein complex system according to previous reports (5). Briefly, the Cas9 nuclease, synthetic single-guided RNAs for IDH1 (5'-TGACGTGCAGTCGGACTCTGTGG-3'), and the single-stranded donor oligodeoxynucleotide for IDH1 p.Ser280Phe (S280F mutation: 5'-ACTACCCTGGAATGACCCTGTTCCTACAGGCCAGGCTCCACCTCTGTACCTTGGGCCACAAAGTCCGACTGCACGTCACCATCATAGTTTTACAGGCCCAGATGAAGCCTCCCTCTGATT-3') were transfected into HuCCT1 cells via electroporation-mediated ribonucleoprotein delivery. After 72 hours of incubation, HuCCT1 cells were collected, resuspended, and seeded into 96-well plates for monoclonal culture. The genomic DNA was extracted from GFP-positive cells and amplified using paired primers (forward: 5'-AAGGATGCTGCAGAAGCTATAAAGAAGCATAATGTTGGCGTCA-3'-; reverse: 5'-TTATTGTTATCAAGCTTTGCTCTGTGGGCTAACCCTCTGGTCC-3'), where the amplified PCR products spanning the IDH1 S280F mutation site underwent subsequent Sanger sequencing.

Cell proliferation assays

The luminescence assays and clone formation assays were performed to evaluate the

cellular proliferative capacity of iCCA with gene alterations or drug treatment. As for luminescence assays, luciferase-carrying iCCA cells were digested and resuspended in complete culture medium (2000 cells/100 μL) and plated in 96-well plate (100 μL/well), whose proliferation was determined by luminescence detection. As for drug sensitivity assays, digested iCCA cells were resuspended in complete culture medium (2000 cells/100 μL) and plated in 96-well plate (100 μL/well), whose viability was evaluated by luminescence after treating with drugs for 3 days. The cellular viability data was analyzed to calculate the IC50 values, and the drug combination efficacy was estimated by the Bliss independence model as previously reported. As for clone formation assays, digested iCCA cells were resuspended in complete culture medium (2000 cells/2 mL) and plated in 6-well plate (2 mL/well), whose clone formation ability was detected by crystal violet staining.

Tumor Migration and Invasion Assays

Tumor transwell assays were performed to evaluate the migrative and invasive capacity of iCCA cells. As for tumor migration assays, the lower chamber of the transwell well was filled with 600 μL of fresh complete culture medium (containing 20% FBS), then the digested iCCA cells were resuspended in serum-free culture medium (3-5 × 10^4 cells/200 μL) and plated onto the upper chamber of the transwell well (200 μL/well). After incubation in thermostatic incubator at 37°C for 24 h, the transwell well was fixed by paraformaldehyde, and the tumor migrative capacity was determined by crystal violet

staining and cell-counting under microscope. As for tumor invasion assays, the upper chamber of transwell well was pre-coated with 60-80 μL matrigel (Cat#354234, Corning; diluted by PBS at 1:5) and placed in 37°C humidified chamber for 2 h for gelling, and the subsequent procedures are the same as tumor migration assays in a seeding density of 4-6 $\times 10^4$ cells/200 μL.

Detection of the magnesium content

The freshly resected tissues, including iCCA tumor, normal bile ducts, and peripheral blood from iCCA patients or tumor-bearing mice were washed with 250 mM sucrose and immediately snap-frozen by liquid nitrogen. Then the collected tissues were immersed by 10% sucrose and homogenized by sonication on ice. After centrifugation at 15000g for 15 min at 4°C, the supernatants were collected for detecting free Mg^{2+} content via atomic absorbance spectrophotometry (Agilent Technologies). The readouts were adjusted and normalized by the blank control and corresponding tissue weights.

Detection of tumor apoptosis

After treated by drugs for intended time, the iCCA cells were harvested and stained for apoptotic analysis using Annexin V APC Apoptosis Detection Kit (Cat#62700, Biogems) according to the manufacturer's instructions. Their apoptosis degrees were determined within 1 hour by a flow cytometer (CytoFLEX S, Beckman) and analyzed by FlowJo

software (Version 10.10.0).

Detection of intracellular ROS

The intracellular ROS level of iCCA cells was evaluated by H2DCFDA staining (MedChemExpress, Cat#HY-D0940) and flow cytometry analysis. Briefly, after treated by drugs for intended time, harvested iCCA cells were incubated with H2DCFDA (10 μM) in 37°C incubator for 30 minutes. Then the cells were washed twice with PBS and the fluorescent intensity was detected by a flow cytometer (CytoFLEX S, Beckman) at ex/em of 488/525 nm.

Measurement of αKG content, GSH/GSSG ratio, and $NADP^+$/NADPH ratio

The content of αKG in iCCA tumors was detected by αKG detection kit (Cat#BC5425, Solarbio) following the supplier's instructions. Briefly, the samples were immersed in the working solution for homogenized by tissue homogenizer or ultra-sound sonication on ice. After centrifugation at 12000g for 10 min at 4°C, the supernatants (0.8 mL) were collected to be slightly mixed with the detection solution (0.15 mL). After another centrifugation at 12000g for 10 min at 4°C, the supernatants were collected for measurement in a microplate reader (Spark10M, TECAN) at emission wavelength of 340 nm. The readouts were adjusted and normalized by the blank control and corresponding tissue weights/cell quantity.

The amount of GSH, GSSG and GSH/GSSG ratio in iCCA tumors were measured by GSH/GSSG detection kit (Cat#S0053, Beyotime) according to the manufacturer's protocols. Briefly, before detection, tissue samples should be grinded to powders. Then the grinded tissues or freshly collected cells were immersed by triple volume of working solution and mixed thoroughly. Following incubation at 4℃ for 10 min and centrifugation at 10000g for 10 min at 4℃, the supernatants were collected for measuring the amount of total glutathione by a microplate reader (Spark10M, TECAN) at emission wavelength of 412 nm. As for measuring GSSG amount, the collected glutathione was mixed with the GSH-eliminating solution at a volume ratio of 5:1. After incubation for 60 min at 25℃, the mixture could be detected for the content of GSSG by a microplate reader (Spark10M, TECAN) at emission wavelength of 412 nm. Since one GSSG molecule can be reduced to two GSH molecules, the calculation for the content of GSH is: GSH=Total Glutathione - GSSG×2. The readouts were adjusted and normalized by the blank control and corresponding tissue weights/cell quantity.

The amount of NADPH, $NADP^+$, and $NADP^+$/NADPH ratio in iCCA tumors were measured by $NADP^+$/NADPH detection kit (Cat#S0179, Beyotime) according to the manufacturer's protocols. Briefly, before detection, tissue samples should be grinded to powders. Then the grinded tissues or freshly collected cells were immersed by pre-cooled extraction solution (200μl per 10^6 cells or 20 mg tissues) and mixed thoroughly. Following incubation at 4℃ for 10 min and centrifugation at 12000g for 10 min at 4℃, the

supernatants were collected for subsequent measuring. For measuring the amount of total NADP (NADPH and $NADP^+$), the G6PDH working solution was added and incubated at 37 ℃ in the dark for 10 minutes, after which the chromogenic solution was added and incubated at 37 ℃ in the dark for 10-20 minutes, and the amount of total NADP was detected by a microplate reader (Spark10M, TECAN) at emission wavelength of 450 nm. As for measuring NADPH amount, the sample was water-bath heated at 60 ℃for 30 minutes to degrade the $NADP^+$, and the following procedures were the same as measuring total NADP. The calculation for the content of $NADP^+$ is: $NADP^+$=Total NADP - NADPH. The readouts were adjusted and normalized by the blank control and corresponding tissue weights/cell quantity.

Protein molecular docking

The protein structure of wild-type IDH1 was obtained from the RCSB PDB database (1T09; https://www.rcsb.org/), and AutoDock Vina software was applied to estimate the interactive docking of wild-type IDH1 to GSK321 or ivosidenib. Taking into account Root Mean Square Deviation (RMSD), Radius of Gyration, Distance of centroid evolution, Buried Solvent Accessible Surface Area (Buried SASA), and interaction energy, the stable state trajectory of the complex is selected. The MM-PBSA (Molecular Mechanics Poisson Boltzmann Surface Area) algorithm is applied to calculate the Gibbs Binding Energy (ΔG_{bind}). The interaction patterns were then analyzed using PyMOL software and plotted

in 3D.

Western blot

Western blot was performed to detect the amount of specific protein in iCCA cells. The chemi-luminescence was detected by the E-Blot machine. The primary antibodies used include IDH1 (Cat#A5106, Abclonal), Flag (Cat#ab205606, Abcam), ATP5A (Cat#ab14748, Abcam), pCHK1 (S345) (Cat#ab58567, Abcam), total CHK1 (Cat#ab40866, Abcam), γH2AX (Cat#9718S, Cell Signaling Technology), total H2AX (Cat#7631S, Cell Signaling Technology), and β-actin (Cat#4970S, Cell Signaling Technology). The β-actin protein served as the internal reference for total protein, and ATP5A protein served as the internal reference for mitochondrial protein. The density of blots was quantified using ImageJ Software (Version 1.54d).

Protein thermal shift assays were performed as previously reported. Briefly, iCCA cells treated by indicated concentration of drugs and $MgSO_4$ were collected by trypsinization. After washing with PBS, resuspending in PBS, lysing through three cycles of snap-frozen by liquid nitrogen and thawing at 37°C, the proteins were heated at the intended temperature (55°C, 59°C, or 63°C) for 3 min. Then the tubes underwent centrifugation at 12000g for 10 min at 4°C to remove denatured proteins, and the supernatants were loaded for immunoblots.

Immunohistochemistry staining

Freshly resected tumors from iCCA patients or tumor-bearing mice were fixed with 10% formalin and then embedded in paraffin and sectioned at 6~8 μm thickness. The histopathological sections underwent dewaxing, rehydration, endogenous peroxidase quenching, membrane penetrating, antigen retrieval, antigen blocking and incubation with primary antibodies to corresponding proteins (IDH1, Cat#A5106, Abclonal; Ki67, Cat#ab15580, abcam; cleaved caspase-3, Cat#9661S, Cell Signaling Technology; pCHK1 (S345), Cat#ab58567, Abcam; γH2AX, Cat#9718S, Cell Signaling Technology; 8-OHdG, Cat#SC-66036, Santa Cruz; 4-HNE, Cat#68538, ProteinTech). Then the sections were treated with DAB peroxidase substrate kit (Cat#K5007, DAKO) and counterstained with hematoxylin (Cat#G1080, Solarbio) for completion of immunohistochemistry (IHC) staining. The protein expression level, defined as the H-score, was determined by grading the staining intensity (0: negative; 1: weak; 2: moderate; 3: strong) and positive area. The protein positive proportion was defined as the ratio of the amount of positive tumor cells to total tumor cells. For IHC analysis, at least three representative regions of interest from individual section were chosen for evaluation.

In the evaluation of IDH expression levels in iCCA tumors before and after GEM-based chemotherapy, we screened iCCA patients from our center who had undergone (i) curative-intent resection, (ii) adjuvant GEM, (iii) postoperative local recurrence, and (iv) a second surgical resection on recurrent iCCA lesions. The retrieved formalin-fixed paraffin-

embedded specimens from the primary and recurrent tumors from the pathology department were used for IDH1 IHC staining.

Immunofluorescence staining

Immunofluorescence staining was conducted according to the supplier's protocols of Tyramide Signal Amplification (TSA) fluorochrome kit (Cat#abs50037, Absin). Briefly, the tissue sections sequentially underwent dewaxing, rehydration, endogenous peroxidase quenching, membrane penetrating, antigen retrieval, antigen blocking, incubation with primary antibodies (IDH1, Cat#A5106, Abclonal; CK19, Cat#ab52625, abcam), incubation with HRP-conjugated secondary antibodies, and incubation with corresponding TSA fluorochrome. Each staining step began from antigen-blocking through TSA fluorochrome incubation. The tissue sections with completed multiplex immunostaining were then analyzed in confocal fluorescence microscope (LSM800, Zeiss) or pathology imaging platform (Vectra Polaris, Akoya).

Magnesium ion fluorescent probing

Magnesium ion fluorescent probing was conducted to evaluate the spatial distribution of magnesium ion in iCCA tumor and normal bile duct histopathological sections according to the manufacturer's instructions (Mag-Fluo-4 AM probe, Cat#MX4544, MKBio). Briefly, histopathological frozen sections underwent formalin fixation and temperature equilibrium

at 37°C for 10 min. Then the working solution of Mag-Fluo-4 AM probe was dripped onto the tissue for incubation at 37°C for 30 min. After washing by PBS thrice, perform the incubation at 37°C for another 30 min. The completed magnesium ion fluorescent probing was then analyzed in confocal fluorescence microscope (LSM800, Zeiss) or pathology imaging platform (Vectra Polaris, Akoya) at emission wavelength of 516 nm.

Metabolic flux

Metabolites of iCCA cells were prepared for GC/MS analysis following a previously published protocol: Cells were ground in liquid nitrogen and resuspended in 1 mL of cold (−40°C) 50% aqueous methanol. The samples were then placed on dry ice for 30 minutes, followed by thawing on ice. Next, 0.4 mL of chloroform was added, and the samples were vortexed for 30 seconds. The mixture was then centrifuged at 14,000 rpm for 15 minutes at 4°C. The supernatant was transferred to new 1.5-mL tubes and evaporated. The samples were stored at −80°C until further analysis. For derivatization, 70 μL of O-isobutylhydroxylamine hydrochloride was added to the dried pellet and incubated for 20 minutes at 85°C. After cooling, 30 μL of *N*-*tert*-butyldimethylsilyl-*N*-methyltrifluoroacetamide (MTBSTFA) was added, and the samples were re-incubated for 60 minutes at 85°C. The samples were then centrifuged at 12,000 rpm for 15 minutes at 4°C. The supernatant was transferred to an autosampler vial for GC/MS analysis.

A Shimadzu QP-2020 GC-MS was programmed with an injection temperature of 250°C,

and 3 μL of sample was injected. The GC flow rate with helium carrier gas was set at 0.92 mL/min. The GC column used was a 30 m × 0.25 mm × 0.25 μm DB-5ms column. The GC-MS interface temperature was 300°C, and the electron impact ion source temperature was set at 200°C, with an ionization voltage of 70V. The mass spectrometer was set to scan the m/z range of 50 to 700, with a detector voltage of 1kV.

The GC/MS data were analyzed to determine isotope labeling and the quantities of metabolites. To assess ^{13}C labeling, the mass distribution for known fragments of metabolites was extracted from the appropriate chromatographic peaks. These fragments included either the entire carbon skeleton of the metabolite or the backbone minus the alpha carboxyl carbon. For each fragment, the data included mass intensities for the lightest isotopomer (M0, without any heavy isotopes) and isotopomers with increasing unit mass (up to M6) relative to M0. These mass distributions were normalized by dividing by the sum of M0 to M6 and corrected for the natural abundance of heavy isotopes of the elements C according to the matrix-based probabilistic methods.

Experimental animals

Immuno-deficient BALB/c nude mice (male, 4-5 weeks old) and severe immuno-deficient NCG mice (male, 4-5 weeks old) were purchased from Zhuhai BesTest Bio-Tech (Guangdong, China). These mice were maintained in the Animal Facilities of South China University of Technology under specific pathogen-free conditions. Ethical approval

involving experimental mice was obtained from the Institutional Animal Care and Use Committee of South China University of Technology (2023071). Animal experiments conform to the Animal Research: Reporting of In Vivo Experiments (ARRIVE) guidelines.

Orthotopic iCCA tumor mice model

In orthotopic iCCA tumor mice models (liver subcapsular tumor inoculation models), 1×10^6 iCCA cells were resuspended in a saline and Matrigel mixture (1:1) in a total volume of 25 μL and then orthotopically inoculated into the liver subcapsular space of 4-week male nude mice. Tumors were first allowed to grow for approximately 3 weeks, then the mice were randomly grouped and treated with different drugs.

For the combination efficacy experiments of GEM and GSK321, the groups include: vehicle, GEM, GSK321, or their combination. The drug therapy was performed for 3 weeks: GEM was intraperitoneally injected at a dose of 10 mg/kg every two days, GSK321 was intraperitoneally administered at 50 mg/kg every day, and the combination was administered with the same dose. Before completion of the treatment, the proliferation of orthotopic tumors was determined using IVIS bioluminescence imaging. Then the mice were euthanized and the tumors were harvested for paraffin-embedding and IHC staining for Ki67 and c-caspase3, aiming to evaluate the alterations in proliferation and apoptosis.

For the in-vivo redox mechanism experiments of GEM and ivosidenib, the groups include: vehicle, GEM, ivosidenib, or their combination. The drug therapy was performed once:

GEM was intraperitoneally injected at a dose of 10 mg/kg, ivosidenib was intraperitoneally administered at 50 mg/kg, and the combination was administered with the same dose. At 24 hours after the therapy, the tumors were harvested and dichotomized, one part of which was collected for detecting the GSH/GSSG and $NADP^+$/NADPH, and the other part was collected for paraffin-embedding and IHC staining for 4-HNE, 8-oxo-dG, pCHK1, and γH2AX, aiming to evaluate the acute alterations in anti-oxidative stress, replication stress and DNA damage.

Patient-derived xenograft (PDX) model

The patient-derived xenograft (PDX) models of iCCA tumors were established in severe immuno-deficient NCG mice to evaluate the therapeutic efficacy of GEM and ivosidenib in human iCCA tumors. Briefly, the freshly resected tumors from iCCA patients were cut into blocks of approximately 5 mm in diameter and then subcutaneously implanted into the NCG mice (P1 generation). When reaching the volume of about 500 mm^3, the P1 tumors were excised, sectioned, and subcutaneously implanted into naïve NCG mice (P2 generation). When the tumor volume reached about 100 mm^3, the P2 mice were randomly grouped and treated with different drugs (vehicle, GEM, ivosidenib, or their combination). The drug therapy was performed for 3 weeks: GEM was intraperitoneally injected at a dose of 10 mg/kg every two days, ivosidenib was intraperitoneally administered at 50 mg/kg every day, and the combination was administered with the same dose. After completion of

the treatment, the mice were euthanized and the tumors were harvested for volume measurement (volume = length × width^2/2) and paraffin-embedded for immunochemistry staining.

Statistical analysis

Clinic-pathological characteristics of iCCA patients or iCCA tumors were compared using $\chi 2$ tests or Fisher exact test for categorical variables, unpaired t-test or one-way analysis of variance (ANOVA) for continuous variables, and Spearman correlation for continuous variables versus continuous variables. Kaplan-Meier estimated survival analysis for OS and the log-rank test were applied to investigate the prognostic association of specific features. Univariate and multivariate Cox proportional hazard models were used to identify significant prognostic variables for overall survival. Drug combination efficacy was estimated by the Bliss independence model. For all the in vitro and in vivo experiments, at least three biological replicates were performed under the same conditions. Most of the statistical analyses were performed with R software, version 4.2.3 (https://www.r-project.org/). All data were expressed as means ± S.D. All statistical tests were two-tailed, and a P value <0.05 was considered significant. Details of the statistical analysis methods were provided in corresponding figure legends.

Supplemental Figure Legend

Supplemental Figure 1. Higher IDH1 was associated with the resistance of iCCA to gemcitabine-based chemotherapy.

(**A**) Boxplot illustrating the distribution of sequencing reads in each sample in the CRISPR/Cas9 synthetic lethal screening (n=3 replicates).

(**B**) Heatmap showing the mutual expression correlation between samples in the CRISPR/Cas9 synthetic lethal screening.

(**C**) Western blot detecting the baseline expression of IDH1 in human iCCA cell lines, and β-actin was used as loading control.

(**D**) Sanger sequencing detecting the IDH1/IDH2 mutational status in iCCA cells.

(**E**) Boxplot illustrating the IHC-score of IDH1 expression in paired iCCA samples before and after GEM-based chemotherapy (n=26 pairs). Representative tiles of IDH1 IHC staining are shown above.

(**F**) Western blot detecting the expression of IDH1 in residual human iCCA cell lines after treatment of GEM, and β-actin was used as loading control.

(**G**) Boxplots illustrating the relative expression of IDH1 in residual cancer cells after treatment of GEM from corresponding GEO datasets.

(**H**) Immunofluorescence co-staining for CK19 and IDH1 in iCCA tumors.

(**I**) Forest plots illustrating the overall survival hazard ratio of clinic-pathological factors by univariate and multivariate Cox regression analysis (n=149).

Statistical analysis: (**E**) paired two-sided t-test, (**G**) One-way ANOVA for GSE105083 and two-sided t-test for GSE118197, (**I**) Cox proportional hazard models. Data represent mean ± SEM.

Supplemental Figure 1

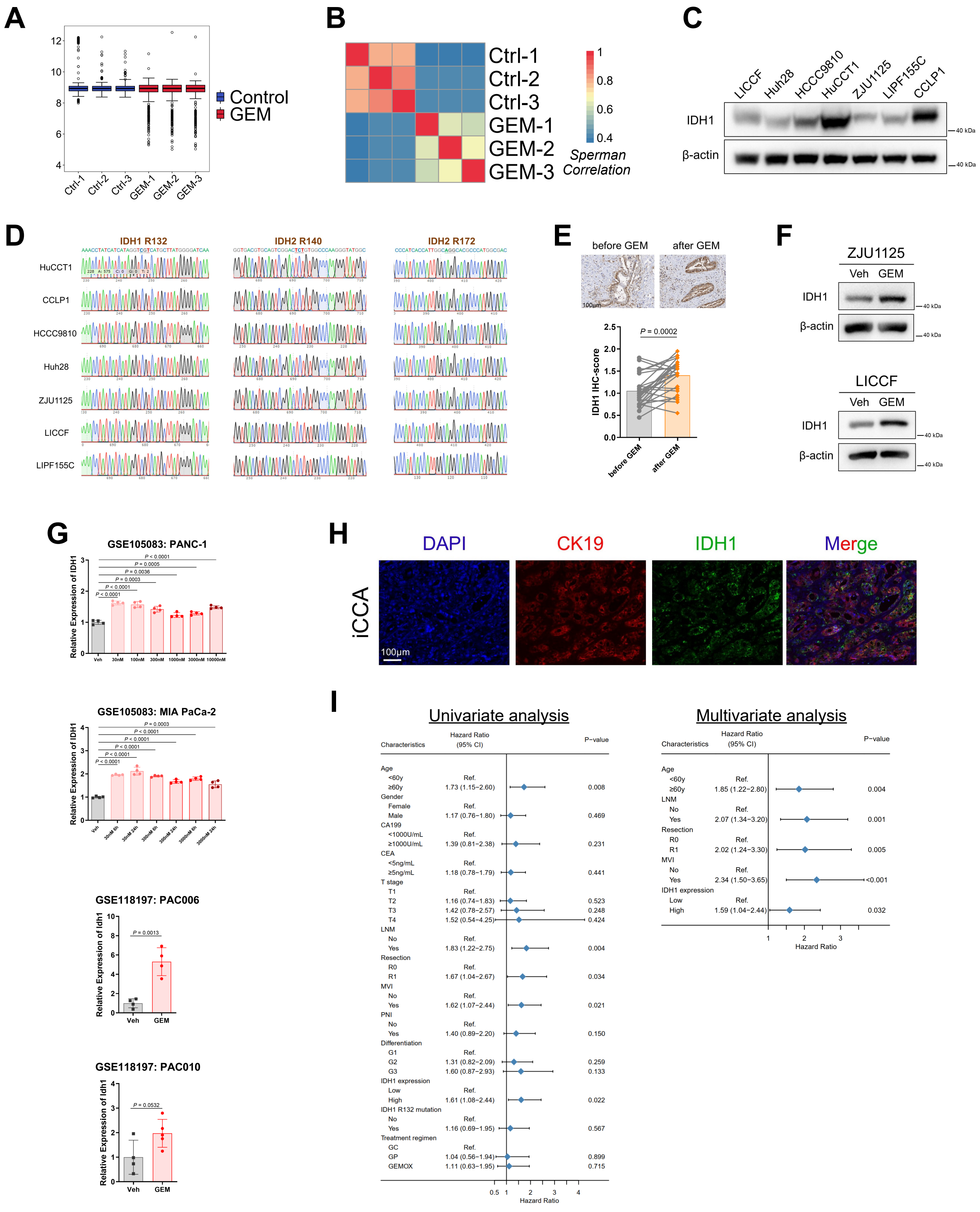

Supplemental Figure 2. Genetic knockdown of IDH1 alleviates the chemoresistance of iCCA to GEM.

(**A-D**) In vitro experiments evaluating the proliferation (**A**), colony-formation (**B**), migration (**C**), and invasion (**D**) capacity of human iCCA cells with altered IDH1 expression.

(**E**) Cellular viability of iCCA cells harboring altered IDH1 expression treated with serial concentrations of GEM for 72 hours.

(**F**) Colony-formation assay of iCCA cells with altered IDH1 treated by GEM (10nM) for 10 days.

Sample size: n=3 replicates for each group. Statistical analysis: (**A-D**, **F**) One-way ANOVA. Data represent mean ± SEM.

Supplemental Figure 2

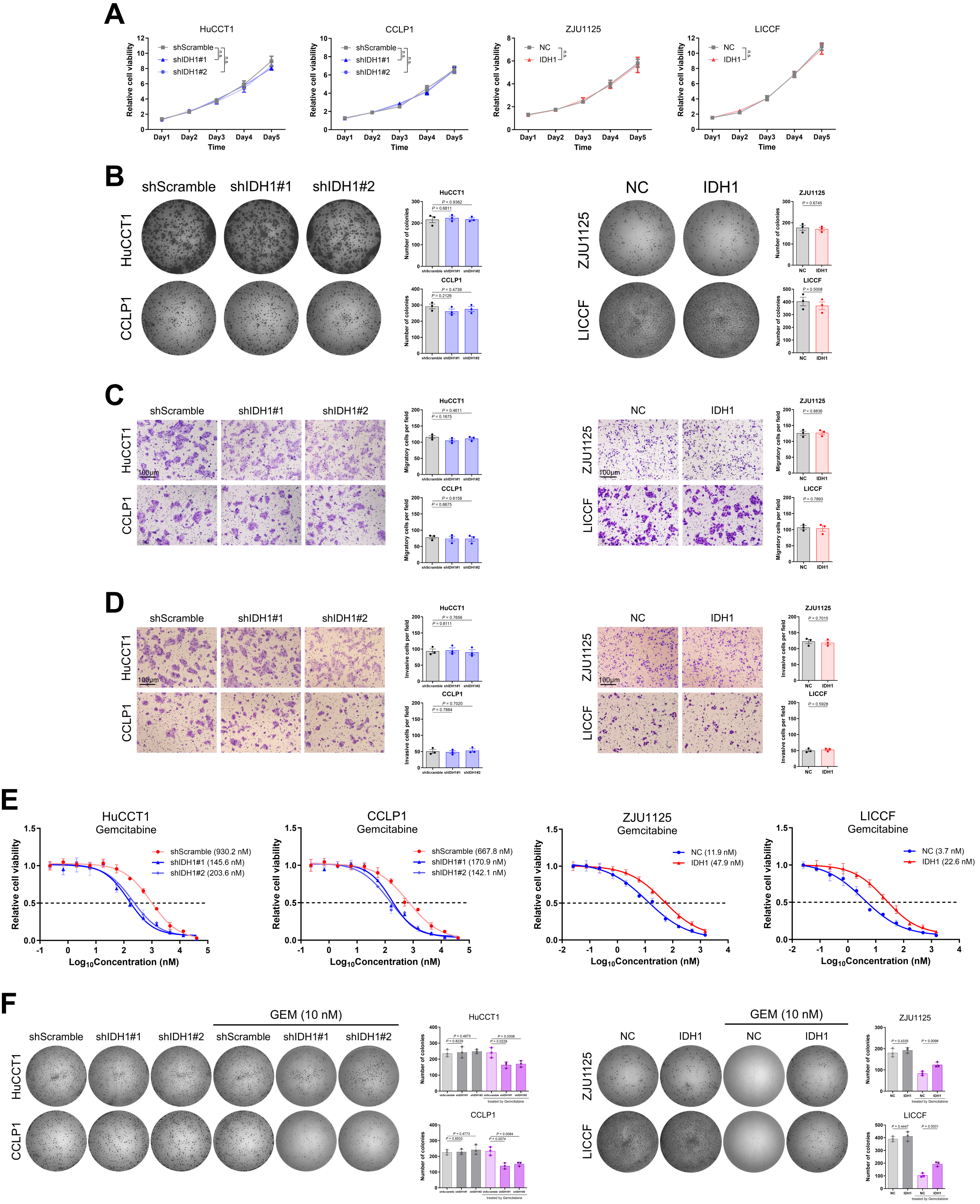

Supplemental Figure 3. Pharmacological inhibition of IDH1 alleviates the chemoresistance of iCCA to GEM.

(**A-D**) In vitro experiments evaluating the proliferation (**A**), colony-formation (**B**), migration (**C**), and invasion (**D**) capacity of human iCCA cells treated by GSK321 (1μM).

(**E**) Cellular viability of iCCA cells treated with GSK321 (1μM) and serial concentrations of GEM for 72 hours, and the calculated IC50 are in brackets.

(**F**) Colony-formation assay of iCCA cells treated with GSK321 (1μM) and GEM (10nM) for 10 days.

(**G**) Colony-formation assay of iCCA cells (after knockdown of IDH1) treated with GSK321 (1μM) and GEM (10nM) for 10 days.

(**H**) Body weights of mice harboring liver subcapsular inoculated HuCCT1 xenografts.

(**I**) IHC staining of Ki67 proliferation in HuCCT1 xenografts.

(**J**) IHC staining of cleaved caspase-3 apoptosis in HuCCT1 xenografts.

Sample size: n=3 replicates for each group, except that n=18 for **H-J**. Statistical analysis: (**A, B, F, I, J**) One-way ANOVA, (**C, D**) Two-sided t-test, (**G**) Two-way ANOVA. Data represent mean ± SEM.

Supplemental Figure 3

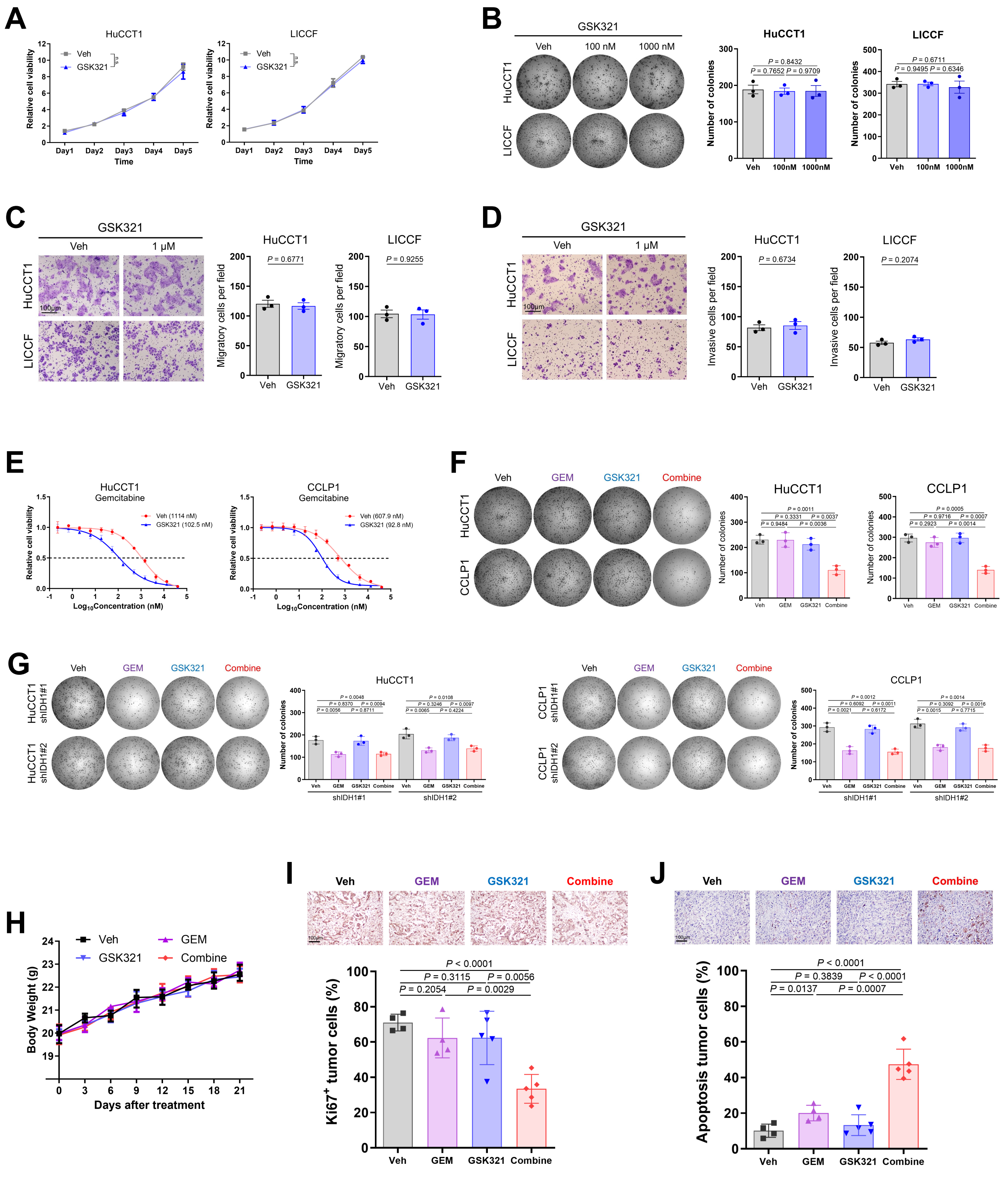

Supplemental Figure 4. Gemcitabine treatment affects intracellular oxidative stress in iCCA.

In vitro assays evaluating the impact of GEM treatment (10nM, 7-10 days) on the proliferation (**A**), oxidative stress (**B**), GSH/GSSG ratio (**C**), and αKG contents (**D**) in iCCA cells.

(**E**) Western blot detecting the expression of pCHK1(S345), total CHK1, γH2AX, and total H2AX in human iCCA cell after treatment of serial concentrations of GEM for 72 hours, and β-actin was used as loading control.

(**F**) Expression ratio of pCHK1(S345) to total CHK1 quantified basing Western blots in human iCCA cell treated with serial concentrations of GEM.

(**G**) Expression ratio of γH2AX to total H2AX quantified basing Western blots in human iCCA cell treated with serial concentrations of GEM.

Sample size: n=3 replicates for each group, except that n=2 replicates for **E-G**. Statistical analysis: (**A-D**) Two-sided t-test. Data represent mean ± SEM.

Supplemental Figure 4

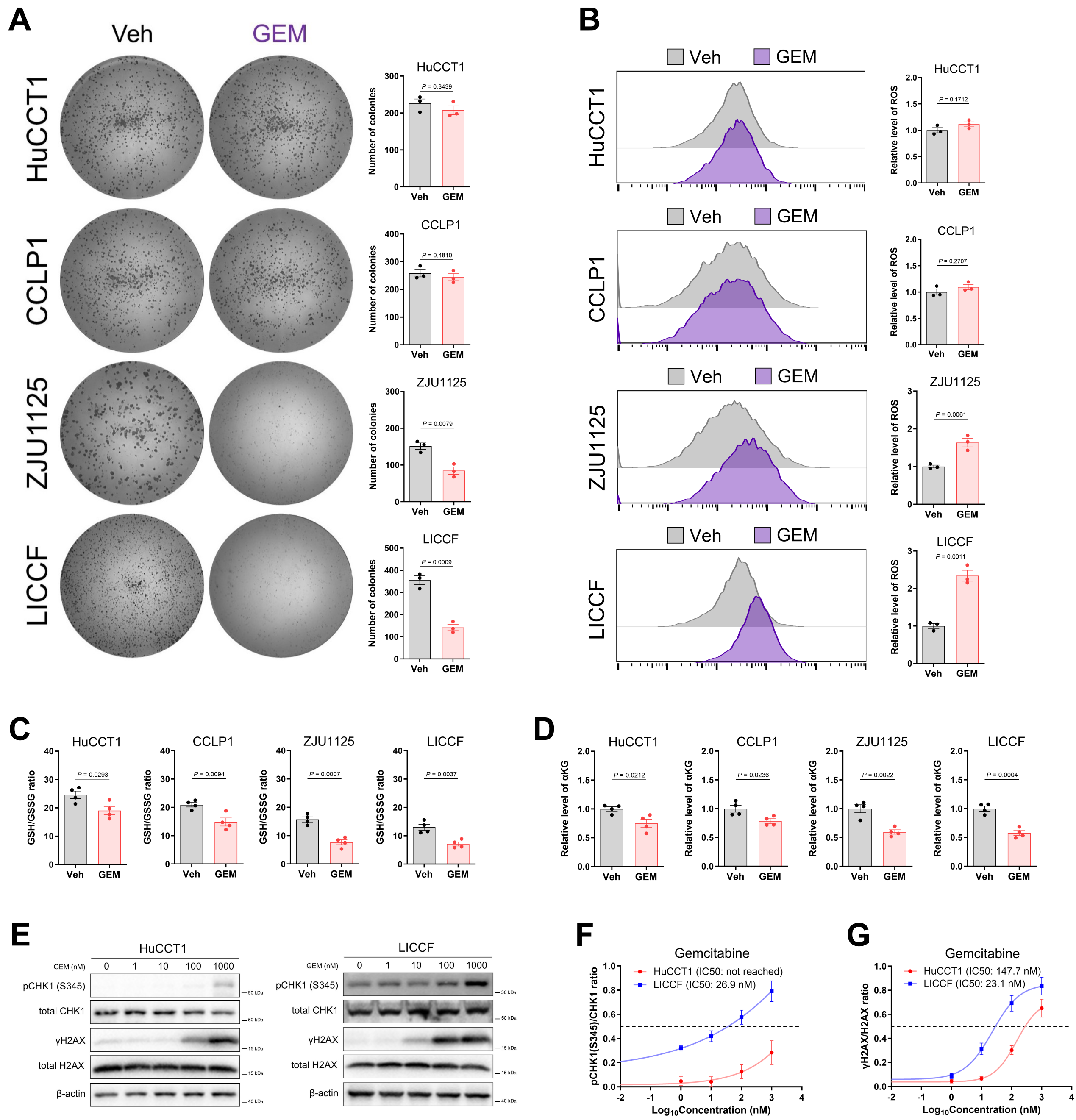

Supplemental Figure 5. IDH1 alteration affects intracellular oxidative stress in iCCA.

(**A-D**) In vitro assays evaluating the rescue effects of overexpressing IDH1 in GEM-sensitive ZJU1125 cells against the impact of GEM treatment (10nM, 10 days) on the proliferation (**A**), oxidative stress (**B**), GSH/GSSG ratio (**C**), and αKG contents (**D**).

(**E-H**) In vitro assays evaluating the synergic effects of combining IDH1 knock-down and GEM treatment (10nM, 10 days) in GEM-resistant CCLP1 cells on the proliferation (**E**), oxidative stress (**F**), GSH/GSSG ratio (**G**), and αKG contents (**H**).

(**I-M**) In vitro assays evaluating the synergic effects of combining IDH1 pharmacological inhibition (1μM) and GEM treatment (10nM, 10 days) in GEM-resistant CCLP1 cells on the proliferation (**I**), oxidative stress (**J**), GSH/GSSG ratio (**K**), and αKG contents (**L**), and $NADP^+$/NADPH ratio (**M**).

Sample size: n=3 replicates for each group. Statistical analysis: (A-M) One-way ANOVA. Data represent mean ± SEM.

Supplemental Figure 5

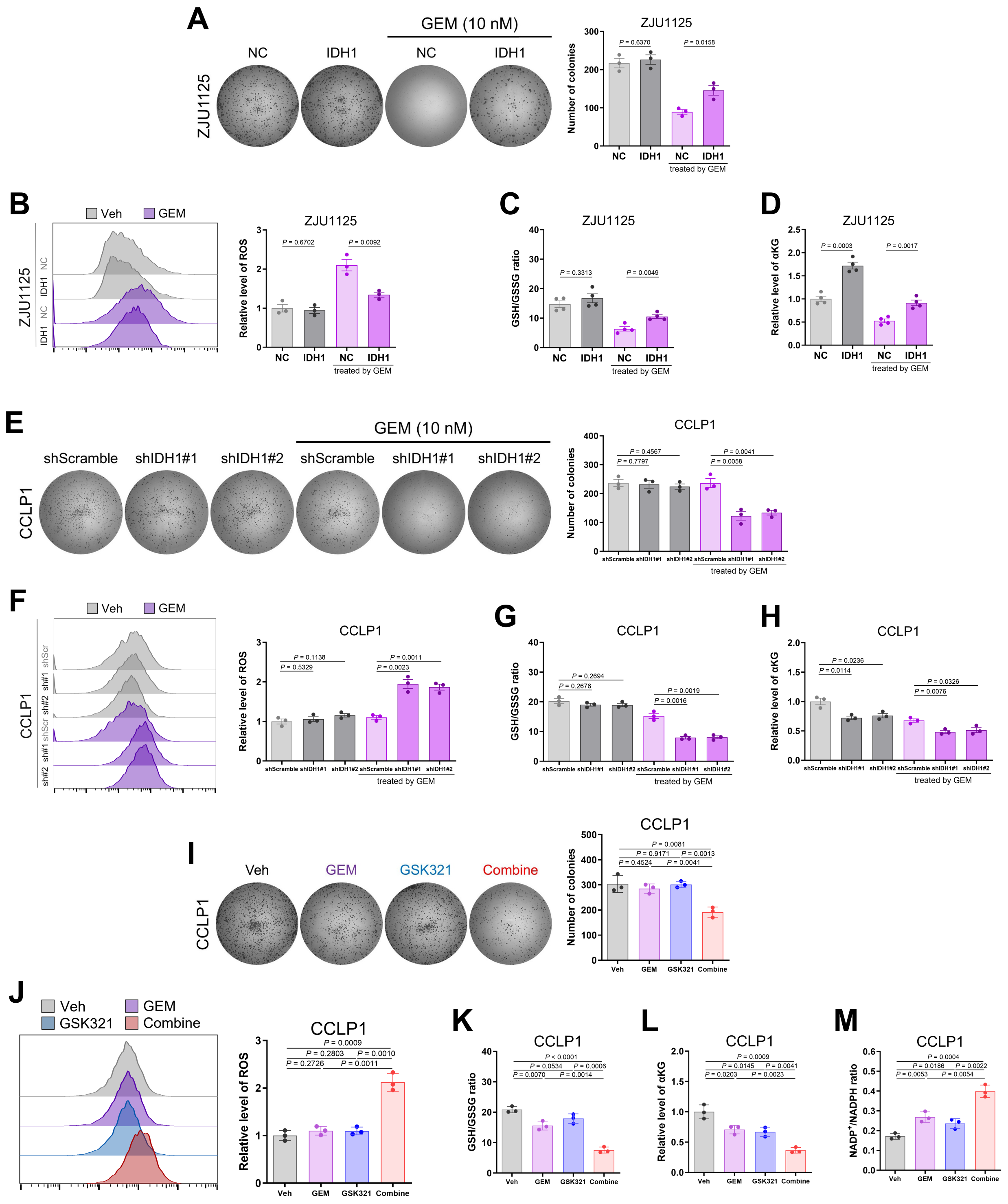

Supplemental Figure 6. The effects of IDH1 in causing resistance of iCCA to GEM depends on its role in maintaining redox homeostasis.

(**A-C**) In vitro assays evaluating the synergic effects of combining pro-oxidants (1% serum starvation, 10μM hydrogen peroxide, 2.5mM glucose withdrawal, and 1μM glutaminase inhibitor CB-839) and GEM treatment (10nM, 10 days) in GEM-resistant CCLP1 cells on the proliferation (**A**), oxidative stress (**B**), and GSH/GSSG ratio (**C**).

(**D-F**) In vitro assays evaluating the rescue effects of supplementing anti-oxidants (1mM N-acetylcysteine, and 4mM glutathione) in GEM-sensitive ZJU1125 cells against the impact of GEM treatment (10nM, 10 days) on the proliferation (**D**), oxidative stress (**E**), and GSH/GSSG ratio (**F**).

(**G-J**) In vitro assays evaluating the rescue effects of overexpressing IDH1 in GEM-sensitive ZJU1125 cells against the synergic efficacy of combining GEM treatment (10nM, 10 days) with pro-oxidants (1% serum starvation, 10μM hydrogen peroxide, 2.5mM glucose withdrawal, and 1μM glutaminase inhibitor CB-839) on the proliferation (**G**), oxidative stress (**H**), GSH/GSSG ratio (**I**), and αKG contents (**J**).

(**K-N**) In vitro assays evaluating the rescue effects of supplementing anti-oxidants (1mM N-acetylcysteine, and 4mM glutathione) in GEM-resistant CCLP1 cells against the synergic efficacy of combining IDH1 knock-down and GEM treatment (10nM, 10 days) on the proliferation (**K**), oxidative stress (**L**), GSH/GSSG ratio (**M**), and αKG contents (**N**).

(**O-R**) In vitro assays evaluating the rescue effects of supplementing anti-oxidants (1mM

N-acetylcysteine, and 4mM glutathione) in GEM-resistant CCLP1 cells against the synergic efficacy of combining IDH1 pharmacological inhibition (1μM) and GEM treatment (10nM, 10 days) on the proliferation (**O**), oxidative stress (**P**), GSH/GSSG ratio (**Q**), and αKG contents (**R**).

Sample size: n=3 replicates for each group. Statistical analysis: (**A-F**, and **O-R**) One-way ANOVA, (**G-N**) Two-way ANOVA. Data represent mean ± SEM.

Supplemental Figure 6

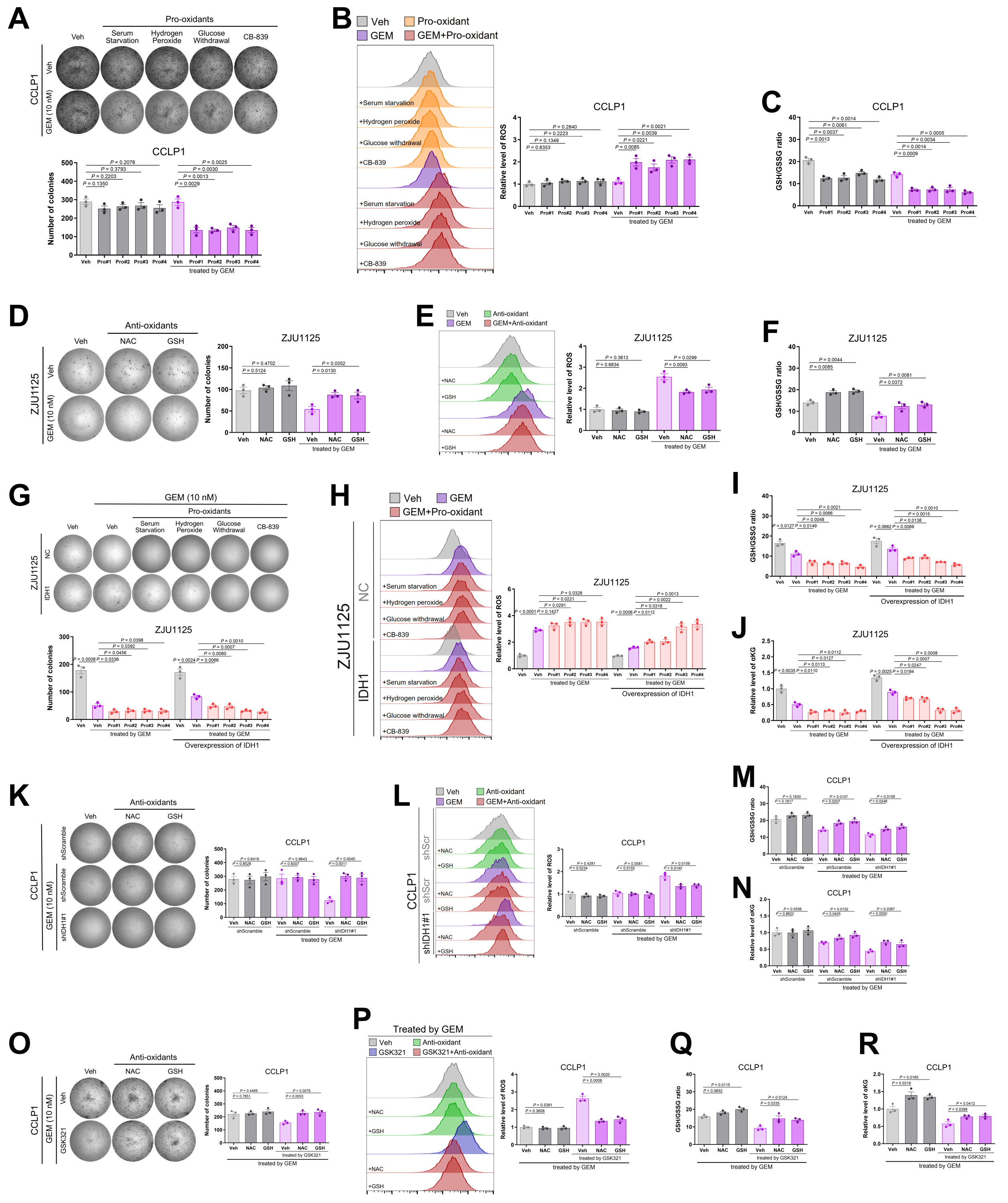

Supplemental Figure 7. Induced NADPH catabolism through introducing mitochondrial TPNOX expression in iCCA cells recapitulates the effects of IDH1 inhibition on GEM sensitivity.

(**A**) Western blot of mitochondrial TPNOX (denoted by anti-Flag) in iCCA cells after 24 hours induction by doxycycline (Dox, 300ng/ml), and ATP5A was used as loading control.

(**B**) $NADP^+$:NADPH ratio measurements in iCCA cells upon Dox induction of mitochondrial TPNOX.

(**C**) $NADP^+$:NADPH ratio measurements in iCCA cells upon mitochondrial TPNOX induction and GEM treatment (10nM, 10 days).

(**D**) Colony-forming assays evaluating the effects of mitochondrial TPNOX induction and GEM treatment (10nM, 10 days) in iCCA cells.

(**E**) $NADP^+$:NADPH ratio measurements in iCCA cells upon mitochondrial TPNOX induction and GSK321 treatment (1μM, 10 days).

(**F**) Colony-forming assays evaluating the effects of mitochondrial TPNOX induction and GSK321 treatment (1μM, 10 days) in iCCA cells.

Sample size: n=3 replicates for each group. Statistical analysis: (**B**) Two-sided t-test, (**C-F**) One-way ANOVA. Data represent mean ± SEM.

Supplemental Figure 7

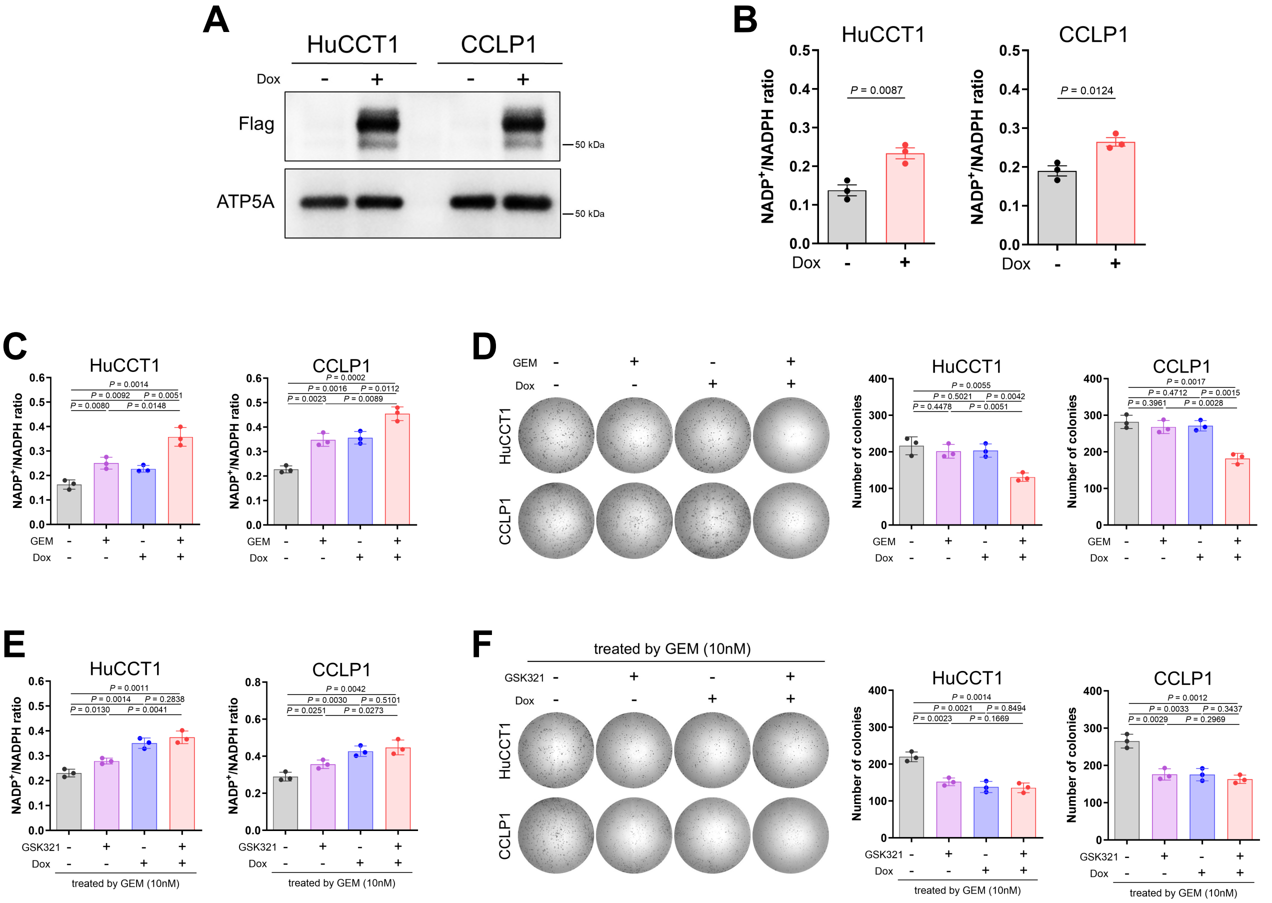

Supplemental Figure 8. Allosteric IDH1 inhibitor ivosidenib shows no significant effects on the malignant characteristics of iCCA under traditional complete medium.

(**A-D**) In vitro assays evaluating the effects of ivosidenib (1μM, 10 days) in iCCA cells on the proliferation (**A**), oxidative stress (**B**), GSH/GSSG ratio (**C**), and αKG contents (**D**) under conventional complete medium.

(**E**) Colony-formation assay of GEM-resistant iCCA cells treated with GEM (10nM) and ivosidenib (1μM) for 10 days under conventional complete medium.

Sample size: n=3 replicates for each group. Statistical analysis: (**A-D**) Two-sided t-test. (**E**) One-way ANOVA. Data represent mean ± SEM.

Supplemental Figure 8

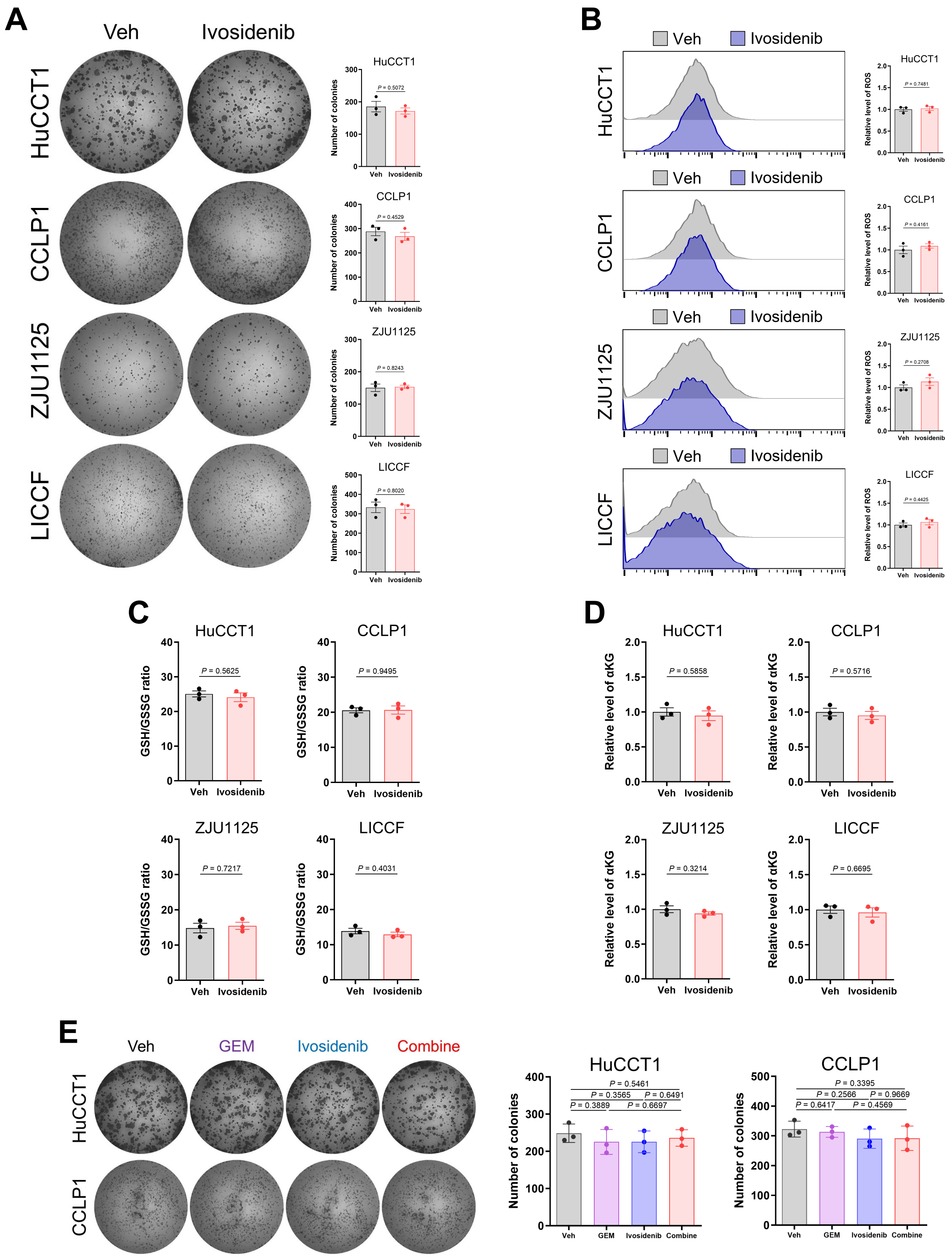

Supplemental Figure 9. Allosteric IDH1 inhibitor ivosidenib inhibits wild-type IDH1 to reverse the resistance of iCCA to GEM.

(**A**) Colony-formation assay of GEM-resistant CCLP1 cells treated with GEM (10nM) and ivosidenib (1μM) for 10 days under magnesium-low complete medium.

(**B**) Relative viability and corresponding Bliss independence scores to the combination of ivosidenib and GEM in GEM-resistant CCLP1 cells under magnesium-low complete medium, where positive values represent synergistic response.

(**C-E**) In vitro assays evaluating the synergic effects of combining ivosidenib (1μM) and GEM treatment (10nM, 10 days) in GEM-resistant CCLP1 cells on the oxidative stress (**C**), GSH/GSSG ratio (**D**), and αKG contents (**E**).

(**F-H**) In vitro assays evaluating the reversal effects of applying ivosidenib (1μM) in GEM-sensitive ZJU1125 cells against the rescue effects of overexpressing IDH1 on the proliferation (**F**), oxidative stress (**G**), and GSH/GSSG ratio (**H**).

(**I-K**) In vitro assays evaluating the rescue effects of supplementing anti-oxidants (1mM N-acetylcysteine, and 4mM glutathione) in GEM-resistant CCLP1 cells against the synergic efficacy of combining ivosidenib (1μM) and GEM treatment (10nM) on the proliferation (**I**), oxidative stress (**J**), and GSH/GSSG ratio (**K**).

(**L-O**) In-vivo orthotopic iCCA tumor model evaluating the 24-hour acute impact of GEM and ivosidenib on lipid peroxidation (**L**; denoted by 4-HNE), DNA oxidation (**M**; denoted by 8-oxo-dG), DNA damage (**N**; denoted by γH2AX and pCHK1), and antioxidant capacity

(**O**; denoted by GSH/GSSG and $NADP^+$/NADPH ratio). Sample size: n=3 replicates for each group, except that n=5 replicates for **L-O**. Statistical analysis: (**A**, **C-E**, **I-O**) One-way ANOVA, (**F-H**) Two-way ANOVA. Data represent mean ± SEM.

Supplemental Figure 9

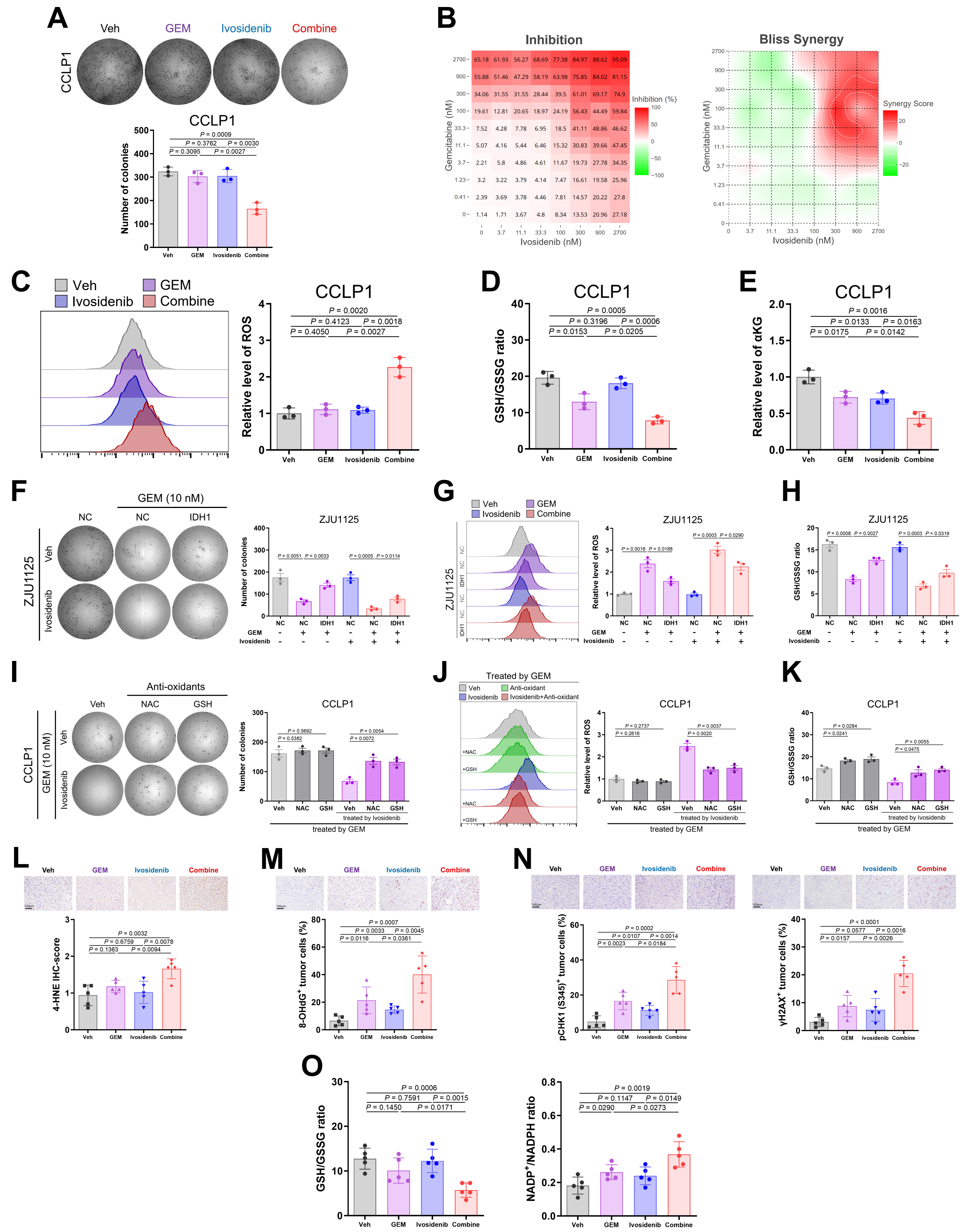

Supplemental Figure 10. Ivosidenib possesses inhibitory effect on wild-type IDH1 in iCCA under physiological tumor microenvironment.

In vitro assays evaluating the impact of serial concentrations of magnesium on the synergic effects of combining ivosidenib (1μM) and GEM treatment (10nM) in GEM-resistant CCLP1 cells on the oxidative stress (**A**), GSH/GSSG ratio (**B**), and αKG contents (**C**). Sample size: n=3 replicates for each group. Statistical analysis: (**A-C**) One-way ANOVA. Data represent mean ± SEM.

Supplemental Figure 10

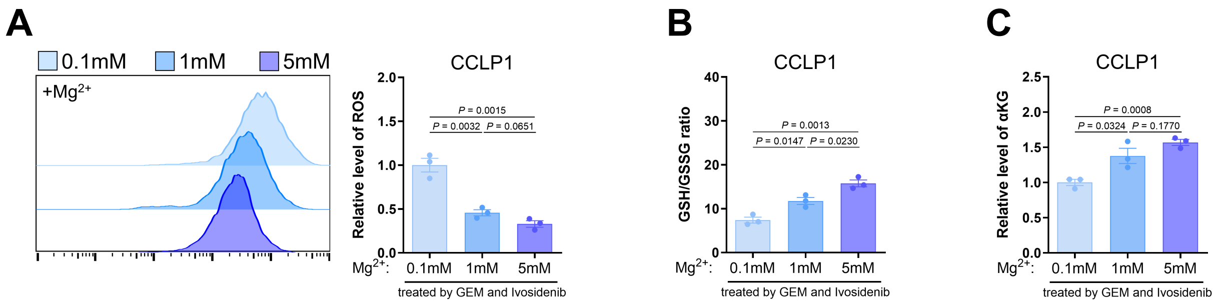

Supplemental Figure 11. Allosteric IDH1 inhibitor ivosidenib synergies with GEM against iCCA with wild-type IDH1 in pre-clinical models.

(**A-B**) IHC staining of Ki67 proliferation (**A**) and cleaved caspase-3 apoptosis (**B**) in iCCA PDX xenografts.

(**C-D**) In vitro assays detecting the contents of GSH/GSSG (**C**) and αKG (**D**) in the lysates of iCCA PDX xenografts after treatment of ivosidenib and/or GEM.

Statistical analysis: (**A-D**) One-way ANOVA. Data represent mean ± SEM.

Supplemental Figure 11

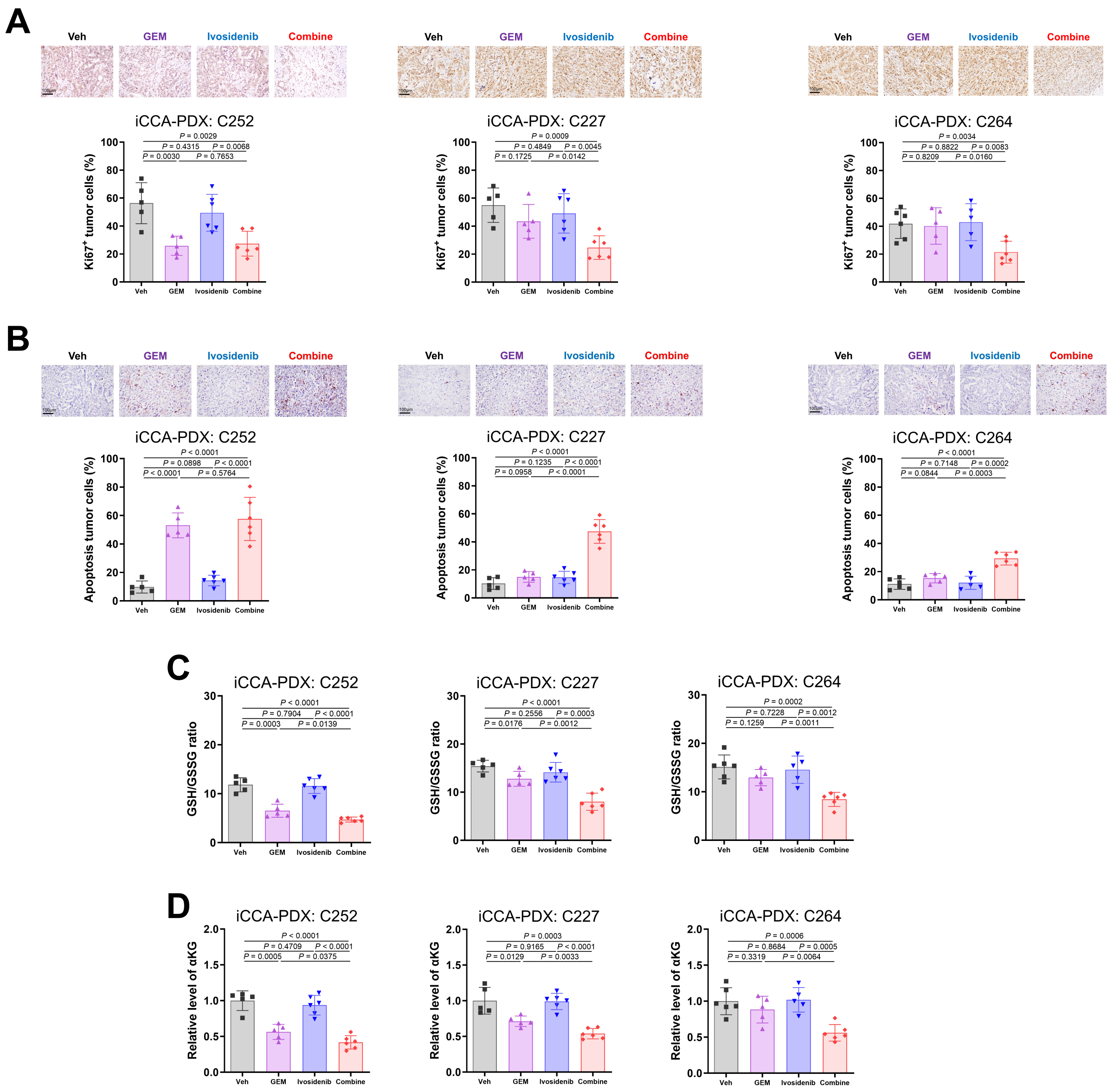

Supplemental Figure 12. The mutation status of IDH1 affects the sensitivity of iCCA cells to GEM treatment and IDH1 inhibition.

(**A**) Sanger sequencing verifying the IDH1 R132S mutation in RBE cells.

(**B**) Luminescence-denoted relative cellular viability of RBE cells treated with serial concentrations of GEM for 72 hours.

(**C**) Colony-forming assays evaluating the effects of olutasidenib (1μM) and GEM treatment (10nM, 10 days) in RBE cells.

(**D-E**) Colony-forming assays evaluating the effects of GSK321 (**D**; 1μM, 10 days) and ivosidenib (**E**; 1μM, 10 days) with GEM treatment in RBE cells.

(**F**) Sanger sequencing verifying the successful introduction of IDH1 S280F mutation into HuCCT1 cells.

(**G**) Colony-forming assays evaluating the anti-tumor effects of combining ivosidenib (1μM) and GEM treatment (10nM, 10 days) in HuCCT1 cells with IDH1 S280F mutation.

(**H**) Western blot evaluating the thermal stability of IDH1 protein in HuCCT1 cells with IDH1 S280F mutation treated by vehicle or ivosidenib (1μM) under culture medium containing indicated concentrations of magnesium.

Sample size: n=3 replicates for each group. Statistical analysis: (**C-E, G**) One-way ANOVA. Data represent mean ± SEM.

Supplemental Figure 12

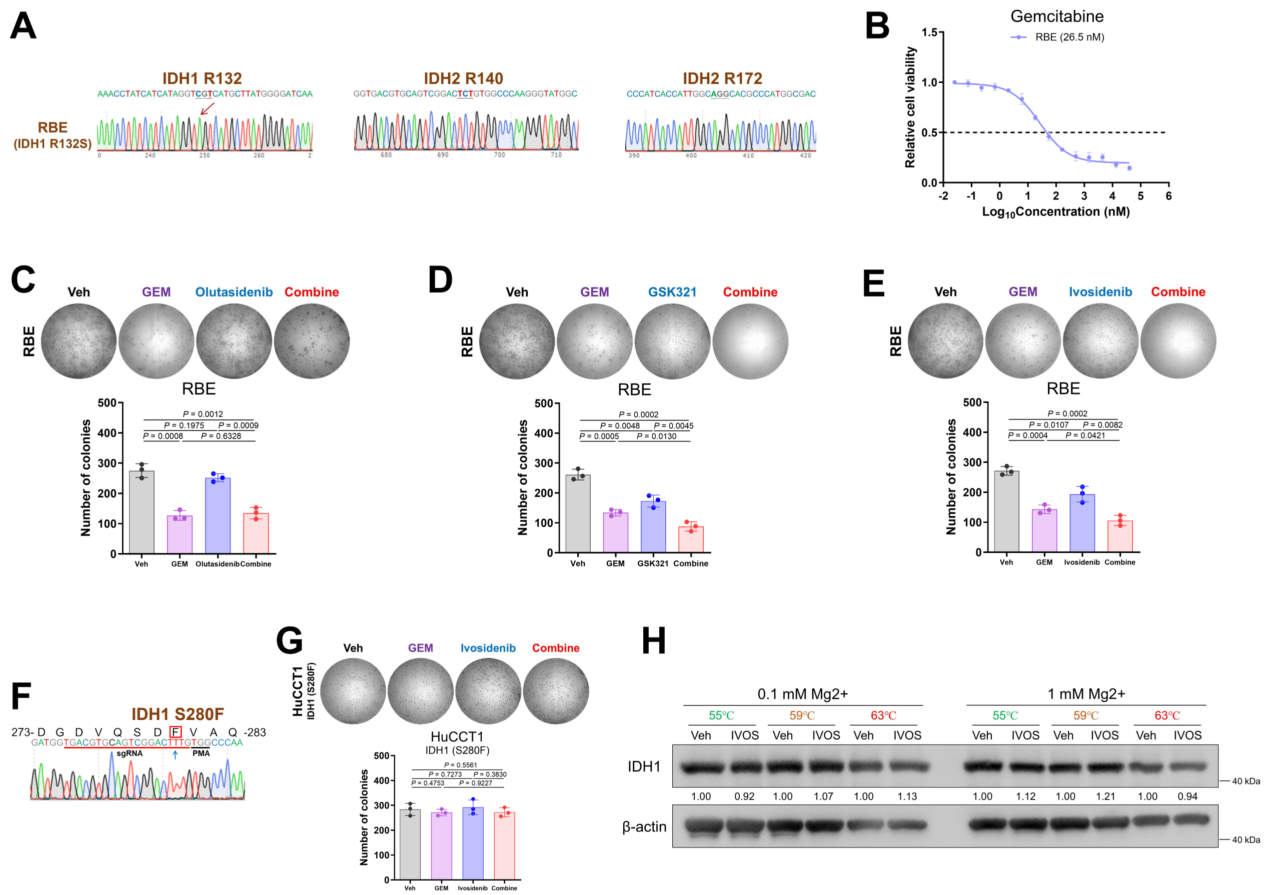

Supplemental Table 1. Clinic-pathological characteristics of enrolled iCCA patients from SYSMH cohort for prognostic Cox regression analysis (n=149).

**Characteristics**	**Number (percentage)**
Age	
<60y	81 (54.4%)
≥60y	68 (45.6%)
Gender	
Female	52 (34.9%)
Male	97 (65.1%)
CA-199	
<1000U/mL	124 (83.2%)
≥1000U/mL	25 (16.8%)
CEA	
<5ng/mL	91 (61.1%)
≥5ng/mL	58 (38.9%)
T stage	
T1	65 (43.6%)
T2	51 (34.2%)
T3	26 (17.4%)
T4	7 (4.7%)
Lymph node metastasis	
No	79 (53%)
Yes	70 (47%)
Resection	
R0	116 (77.9%)
R1	33 (22.1%)
Microvascular invasion	
No	81 (54.4%)
Yes	68 (45.6%)
Perineural invasion	
No	106 (71.1%)
Yes	43 (28.9%)
Histological differentiation	
G1	49 (32.9%)
G2	78 (52.3%)
G3	22 (14.8%)
IDH1 expression	
Low	75 (50.3%)
High	74 (49.7%)
IDH1 R132 mutation	
No	124 (83.2%)
Yes	25 (16.8%)
Treatment regimen	
GC	27 (18.1%)
GP	44 (29.5%)
GEMOX	78 (52.3%)

Supplemental references

1. Jin H, Shi Y, Lv Y, Yuan S, Ramirez CFA, Lieftink C, et al. EGFR activation limits the response of liver cancer to lenvatinib. *Nature.* 2021;595(7869):730-4.
2. Qiao W, Xie X, Shi PY, Ooi YS, and Carette JE. Druggable genome screens identify SPP as an antiviral host target for multiple flaviviruses. *Proceedings of the National Academy of Sciences of the United States of America.* 2025;122(8):e2421573122.
3. Zhang H, Zhu K, Zhang R, Guo Y, Wang J, Liu C, et al. Oleic acid-PPARγ-FABP4 loop fuels cholangiocarcinoma colonization in lymph node metastases microenvironment. *Hepatology (Baltimore, Md).* 2024;80(1):69-86.
4. Cracan V, Titov DV, Shen H, Grabarek Z, and Mootha VK. A genetically encoded tool for manipulation of NADP(+)/NADPH in living cells. *Nature chemical biology.* 2017;13(10):1088-95.
5. Wang J, Yang Y, Shao F, Meng Y, Guo D, He J, et al. Acetate reprogrammes tumour metabolism and promotes PD-L1 expression and immune evasion by upregulating c-Myc. *Nature metabolism.* 2024;6(5):914-32.
