# Supplementary material for: The allosteric IDH1 inhibitor ivosidenib overcomes chemoresistance in intrahepatic cholangiocarcinoma models expressing wild-type IDH1
Source: J Clin Invest. 2026 Mar 17;136(10):e199730. doi: 10.1172/JCI199730 (PMC13178645; doi:10.1172/JCI199730)

Full unedited blots for **Figure 7F**

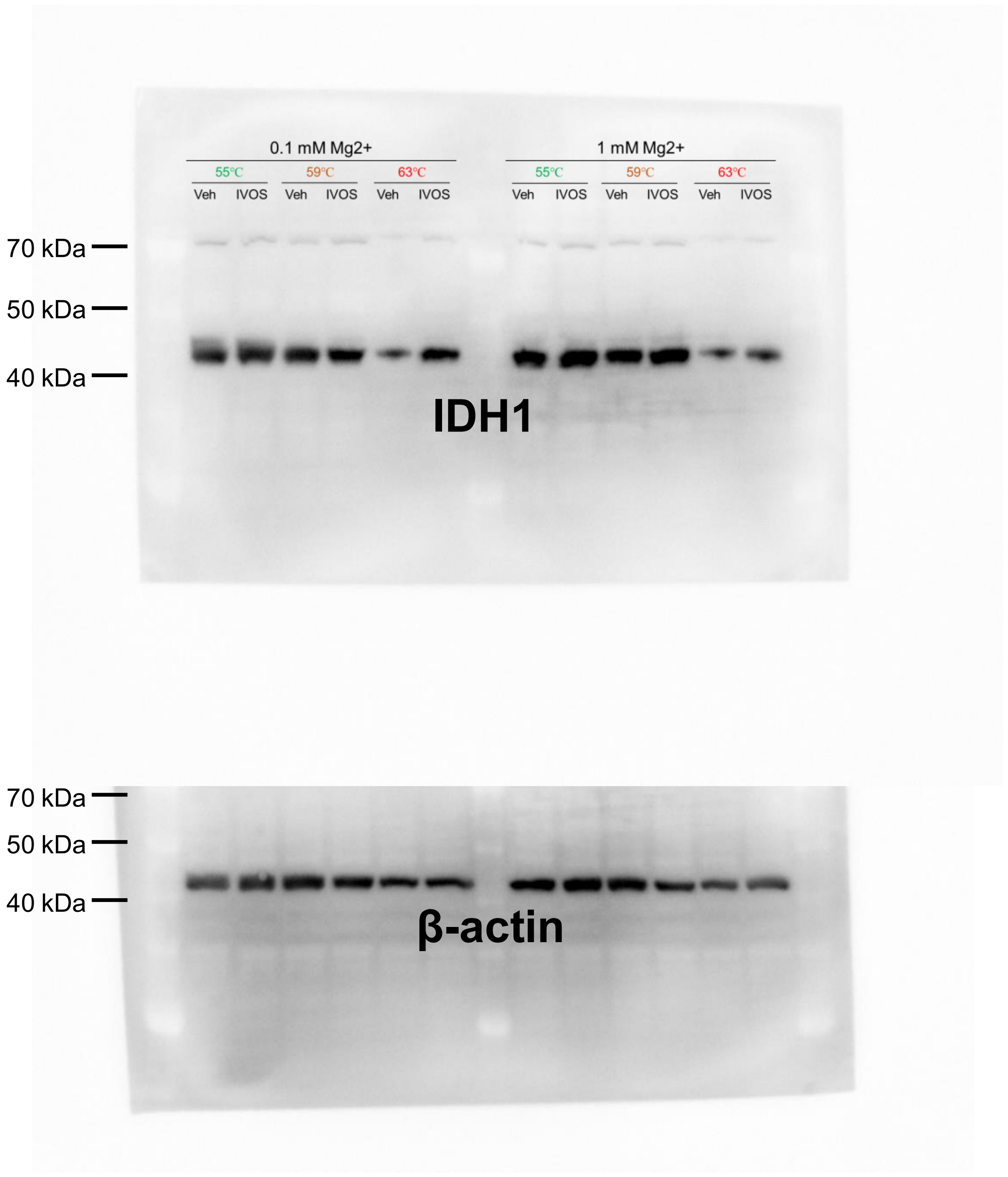

Full unedited blots for **Supplemental Figure 1C**

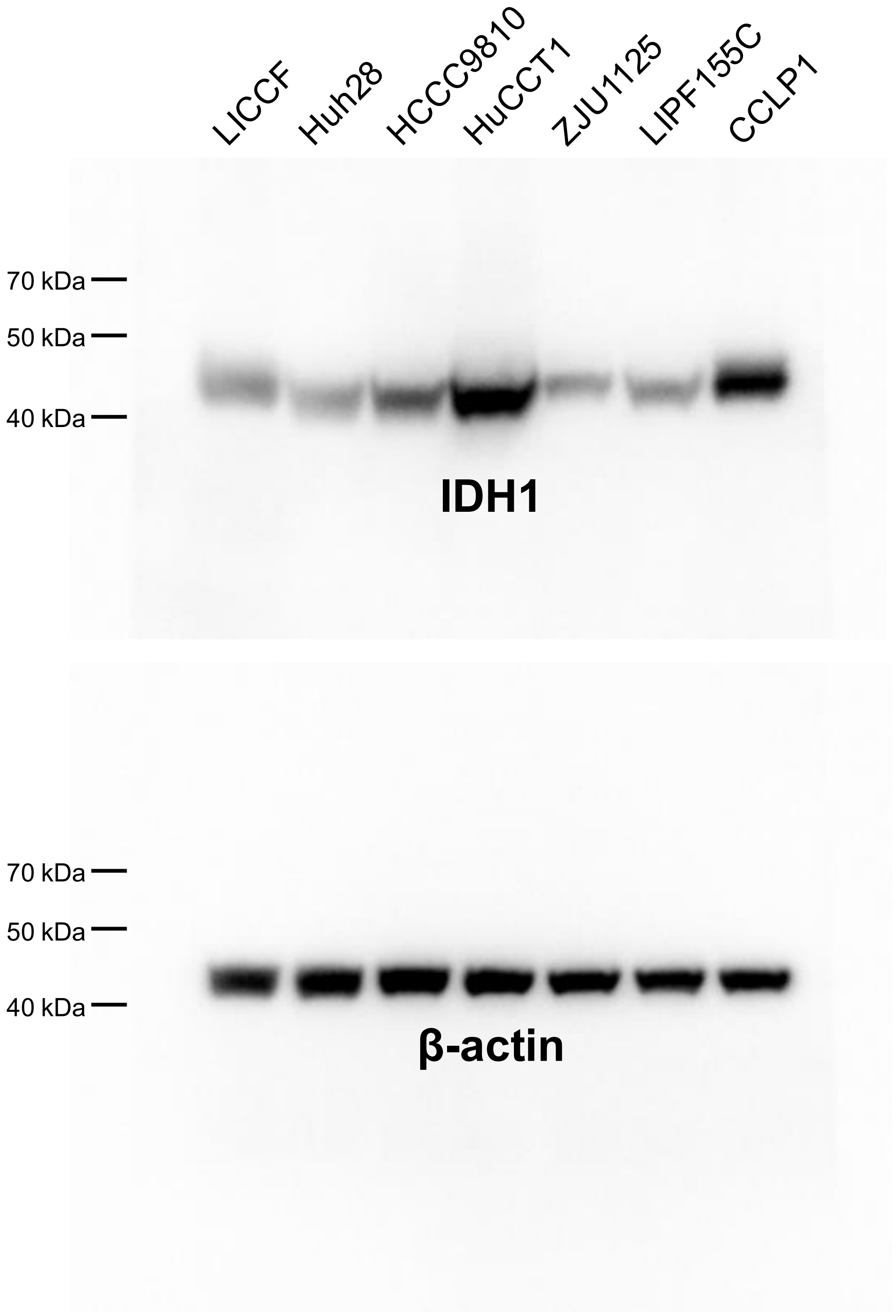

Full unedited blots for **Supplemental Figure 1F**

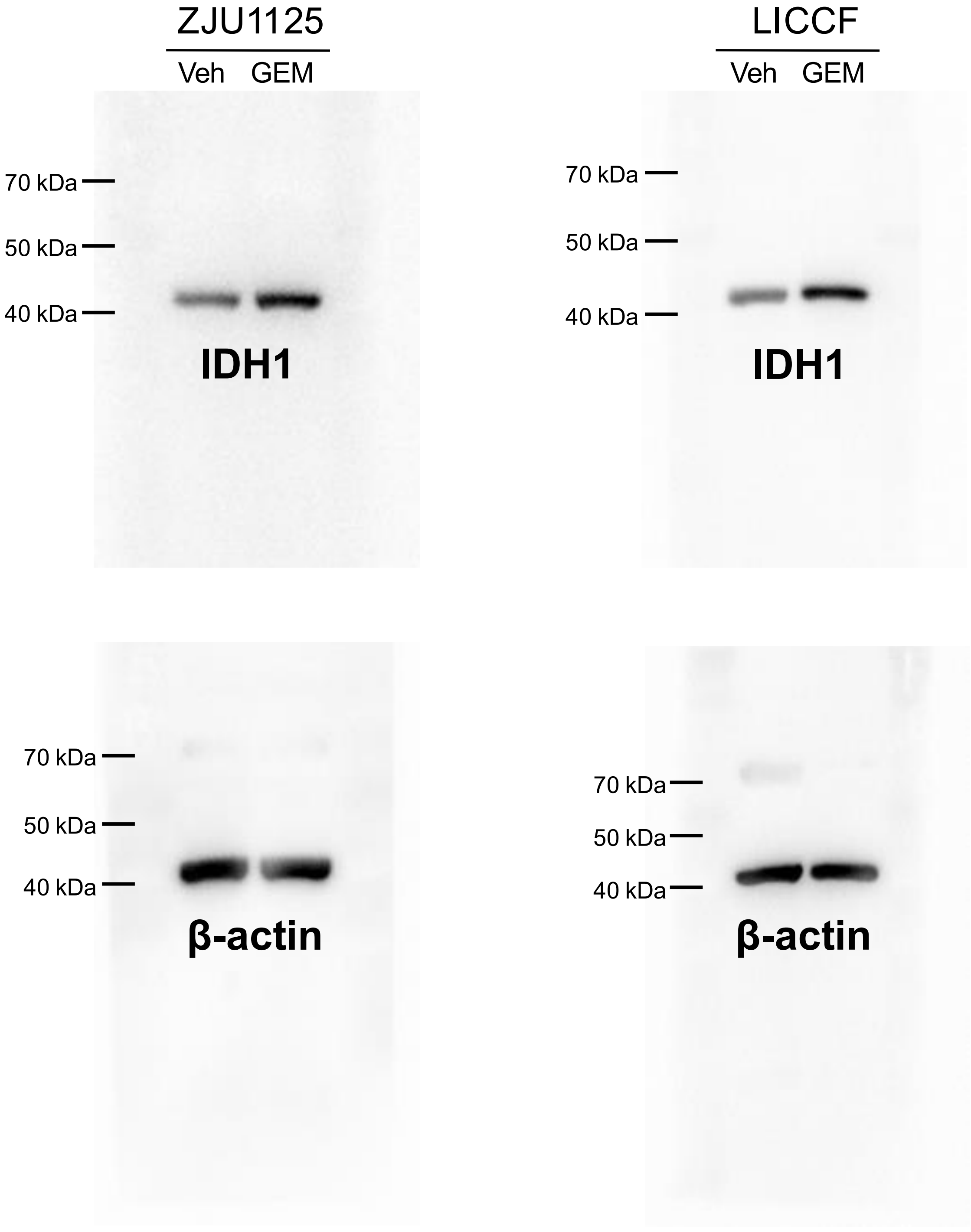

Full unedited blots for **Supplemental Figure 4E**

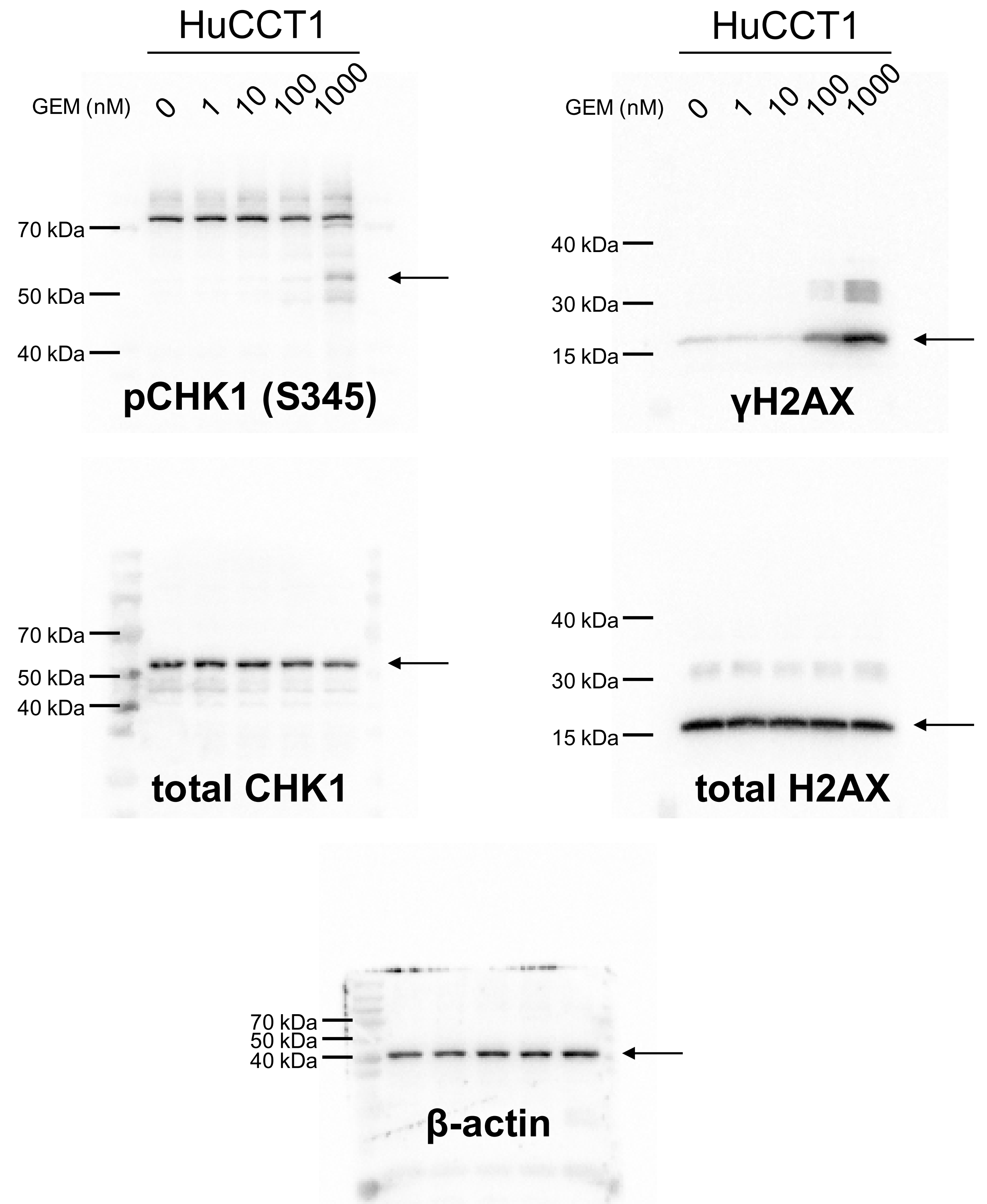

Full unedited blots for **Supplemental Figure 4E**

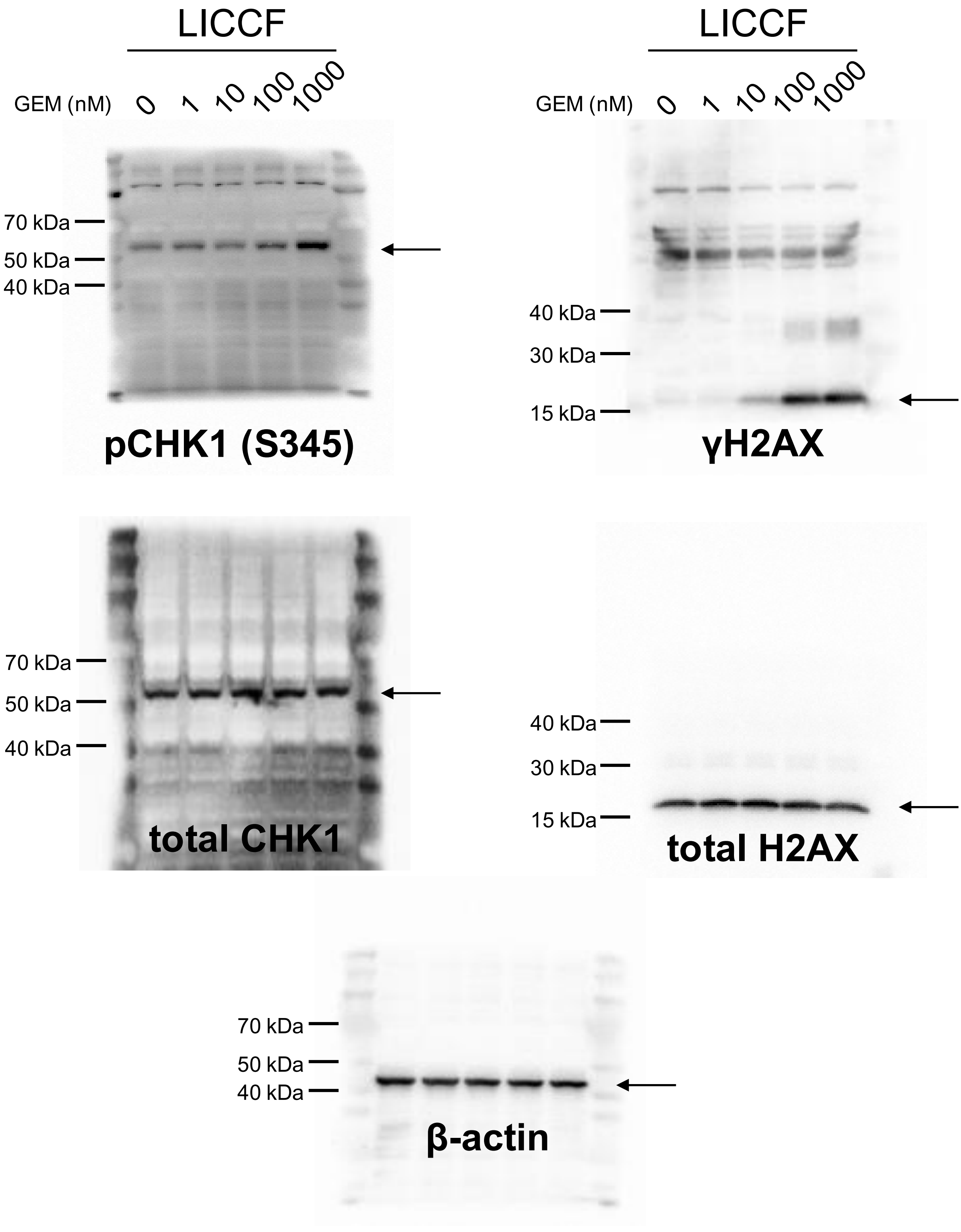

Full unedited blots for **Supplemental Figure 7A**

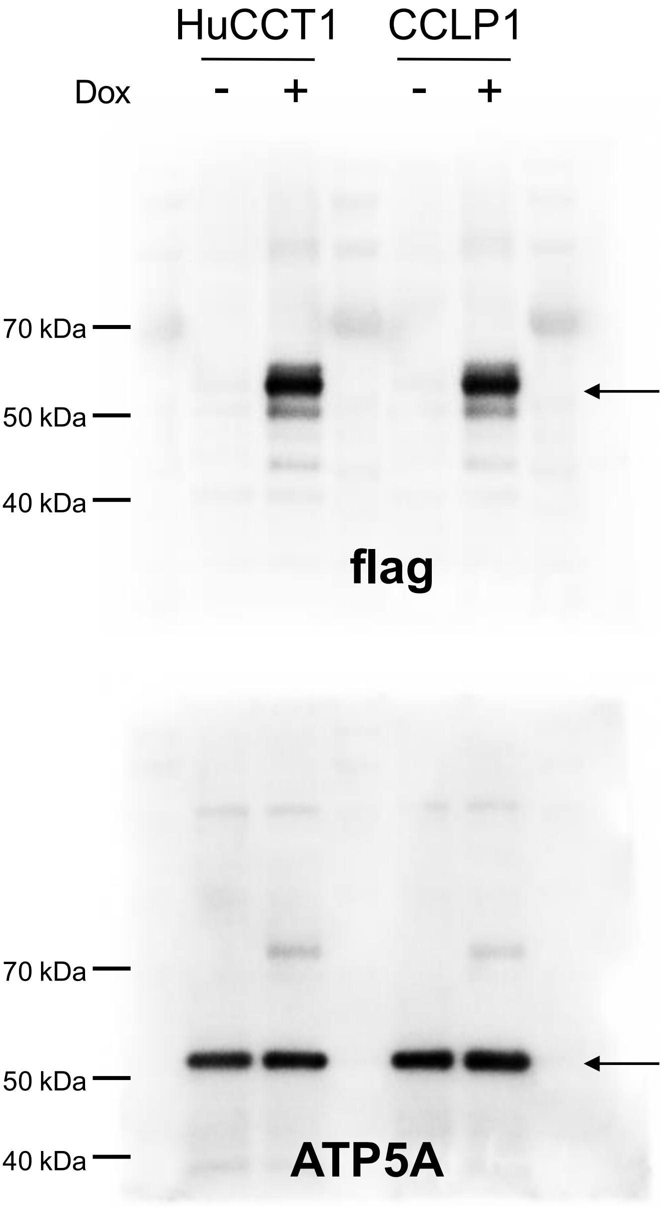

Full unedited blots for **Supplemental Figure 12H**

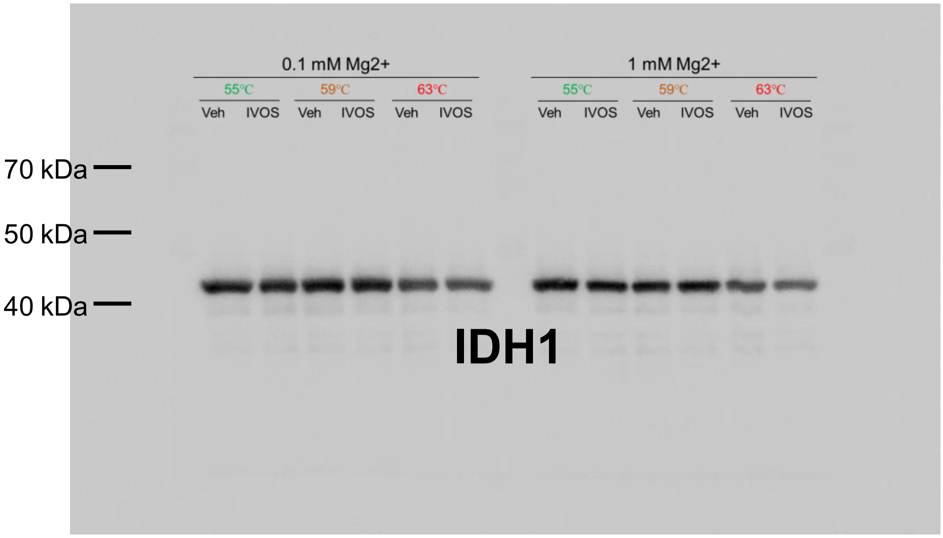

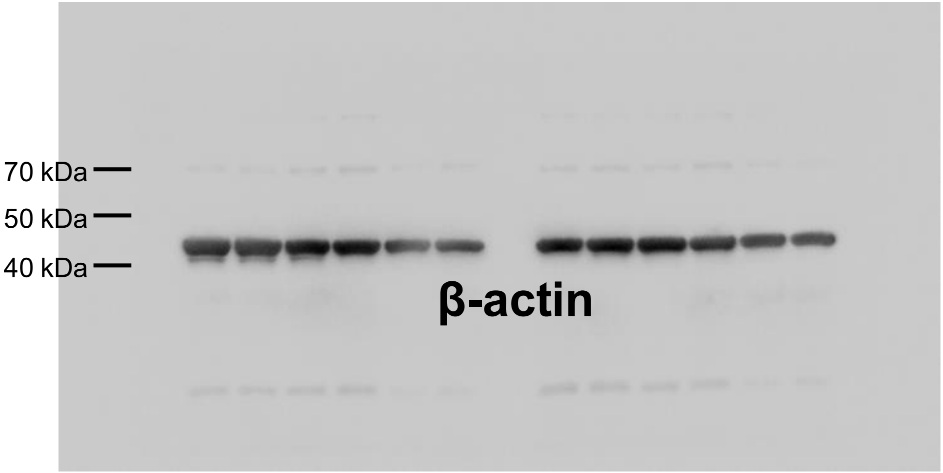

Supplement: Unedited blot and gel images [file jci-136-199730-s216.pdf]
